# Diabetes mellitus and atrophic gastritis: progress in the study of interactive pathogenesis

**DOI:** 10.3389/fendo.2026.1955709

**Published:** 2026-10-21

**Authors:** Zexin Zhu, Ling Wang, Tingting Zhou, Yi Che, Ye Shi, Jiamei Song, Ziyu Zhang, Jian Xu, Shunyuan Tian, Ning Qu, Yihan Lu, Yuxin Hao, Jingzhou Zhang, Hongfei Gao, Guangfu Lv

**Affiliations:** 1 Changchun University of Chinese Medicine, Changchun, China; 2 The Affiliated Hospital of Changchun University of Chinese Medicine, Changchun, China; 3 Changchun Traditional Chinese Medicine Hospital, Changchun, China

**Keywords:** atrophic gastritis, autoimmunity, diabetes mellitus, gastrointestinal hormones, metabolism, oxidative stress, two-way interaction

## Abstract

Diabetes mellitus and atrophic gastritis are two chronic diseases with high incidence, and their comorbidity has attracted increasing attention. The traditional view primarily focuses on the unidirectional damage caused by diabetes to the gastric mucosa, but ignores the possible bidirectional pathological relationship between the two diseases. This article reviews research progress on the reciprocal pathogenesis between DM and the gastric mucosa and posits a theoretical framework of a “bidirectional vicious cycle.” On the one hand, diabetes may promote the occurrence and development of atrophic gastritis through multiple pathways. On the other hand, atrophic gastritis may have a profound reverse effect on glucose metabolism. The “common soil” for the coexistence of the two diseases is also revealed, including shared HLA class II susceptibility genes, Th17/Treg immune imbalance, and ROS-NF-κB-mitophagy disorders coupled with a molecular network. It should be noted that most of the proposed bidirectional framework is based on accumulating preclinical and observational evidence, and large-scale prospective cohort studies are urgently needed to establish causal temporal relationships. Based on the above mechanisms, this paper presents antioxidant-related intervention strategies and identifies the current study’s limitations. The purpose of this review is to promote the new clinical concept of “glucose-gastrotube co-administration” and provide a theoretical basis for breaking the mutually reinforcing cycle between the two diseases and reducing the risk of decompensation in gastric precancerous lesions and diabetes.

## 1 Introduction

Diabetes mellitus and atrophic gastritis are two common chronic diseases that seriously threaten human health. The coexistence of the two diseases in clinical practice is by no means accidental (1). Epidemiological studies have shown that patients with diabetes have gastric mucosa atrophy, intestinal metaplasia, and gastric cancer, and the disease prevalence is significantly higher than in people without diabetes (2). Traditional medical practice, however, for a long time, placed these two diseases within the framework of independence: endocrinologists focus on blood glucose control and complication prevention, and digestive department doctors focus on helicobacter pylori eradication and mucosal protection. The discipline division raises a key scientific problem that has long been neglected: whether the relationship between diabetes and atrophic gastritis extends beyond single-organ damage, and whether there is a two-way pathologic correlation. In other words, are hyperglycemia and its associated metabolic disorders not only bystanders but also active drivers of gastric mucosal atrophy? At the same time, gastric acid deficiency, micronutrient malabsorption, and remodeling of the gastrointestinal hormone network caused by atrophic gastritis will reverse the deterioration of glucose metabolism, thereby forming a hidden “gastrogenic diabetes accelerator” effect.

In recent years, with further advances in medical science, the answer to this question has gradually become clearer. The central thesis of this review is that diabetes and atrophic gastritis may constitute a “bidirectional vicious cycle” driven by the intertwining of multiple molecular events. From the perspective of diabetes promoting atrophic gastritis, long-term hyperglycemia exerts pathological pressure through three core pathways. First, oxidative stress induced by hyperglycemia and accumulation of advanced glycation end products (AGEs) directly damage gastric mucosal epithelial cells, and amplify local inflammation and apoptosis through the NF-κB signaling axis. Second, diabetic microangiopathy and chronic gastric mucosal ischemia caused by autonomic neuropathy, together with hypoperfusion and motility disorders—including the reduction of interstitial cells of Cajal, delayed gastric emptying, and gastric electrical rhythm disturbance—are key mechanisms of bile reflux. Third, hyperglycemia alters the gastric mucosal environment, modifies glycosylation patterns of mucins, weakens the gastric acid barrier, increases susceptibility to Helicobacter pylori and its immune escape ability after infection, and accelerates progression from chronic gastritis to malignant transformation.

On the other hand, the reverse effect of atrophic gastritis on glucose metabolism is equally hidden and critical. The progressive loss of gastric parietal cells, with low or absent gastric acid secretion, first impairs the absorption of vitamin B_12_ and iron, leading to hyperhomocysteinemia and dysfunction of the mitochondrial electron transport chain. The former aggravates peripheral insulin resistance through oxidative stress injury, while the latter directly impairs glucose-stimulated insulin secretion of β-cells. At the same time, the loss of negative feedback inhibition of gastric antral G cells sustains the release of large amounts of gastrin, forming extreme hypergastrinemia, and the reduction of X/A-like cells in the gastric fundus leads to ghrelin deficiency. The extreme imbalance of these two hormones accelerates β-cell depletion through CCK-B receptor overstimulation, rather than providing metabolic benefits. In addition, small intestinal bacterial overgrowth (SIBO) induced by low gastric acid produces large amounts of lipopolysaccharide, which enters the portal vein and causes “metabolic endotoxemia,” continuously activating systemic low-grade inflammation, impairing the incretin axis, and further worsening postprandial glucose control.

Diabetes is more profound, diabetes and shares a set of atrophic gastritis, chronic low-grade inflammation, autoimmune disorders, and mitochondrial dysfunction as the core “common soil”, making two diseases reinforce each other in the same individual, and progress in coordination.

Based on the above problems and the related research background, this review aims to systematically examine the pathological mechanism by which diabetes promotes atrophic gastritis, the molecular pathways by which atrophic gastritis mitigates worsening glucose metabolism, and the shared soil and overlapping mechanisms underlying the coexistence of the two diseases. On this basis, the potential therapeutic targets for the interaction link were refined, the shortcomings of existing studies were evaluated, and the future direction of precise classification and comorbidity management from “sugary-stomach dialogue” was prospected. We expect that by integrating the breaking of the organ-perspective barrier, we can clinically block mutually promoting circulation, reduce pathological changes prior to gastric carcinoma, and provide a new theoretical basis and practical strategy for reducing the risk of end-stage sugar metabolic decompensation.

## 2 The pathological mechanism of diabetes promoting atrophic gastritis

In a diabetic state, chronic hyperglycemia does not damage gastric mucosa through a single pathway, but constitutes a multi-level and multi-target pathological attack network, with hyperglycemic toxicity as the core initiating factor (3). At the same time, microvascular lesions with autonomic neuropathy, causing gastric mucosa of chronic ischemia, low perfusion, and dynamic obstacles, provide atrophic changes continuously organized microenvironmental stress (4). A high-sugar microenvironment enhances Helicobacter pylori engraftment and immune escape, and gastrointestinal hormone imbalance disrupts mucosal repair and the steady state (5). The proposed bidirectional pathological interactions between diabetes and atrophic gastritis are illustrated in **Figure 1**.

**Figure 1:**
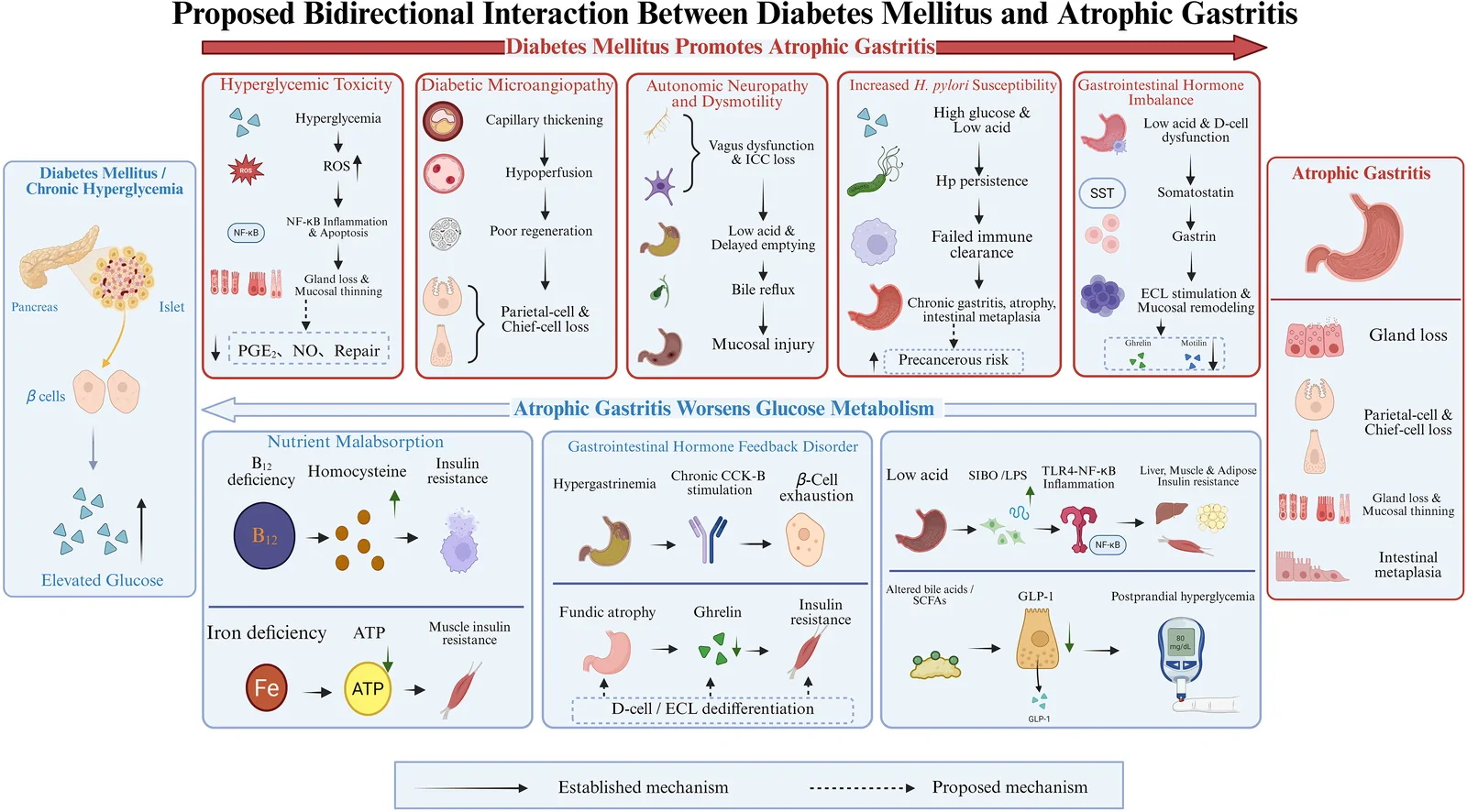
Proposed bidirectional pathological interactions between diabetes mellitus and atrophic gastritis. Diabetes mellitus may promote gastric gland loss, parietal- and chief-cell depletion, and intestinal metaplasia through hyperglycemic toxicity, gastric microangiopathy, autonomic neuropathy and dysmotility, increased susceptibility to Helicobacter pylori, and gastrointestinal hormone imbalance. Conversely, atrophic gastritis may worsen insulin resistance, β-cell dysfunction, and glycemic control through vitamin B12 and iron malabsorption, gastrointestinal hormone dysregulation, and hypochlorhydria-associated dysbiosis with impairment of the incretin axis. Solid arrows indicate established mechanisms supported by clinical or experimental evidence; dashed arrows indicate proposed or incompletely validated pathways. DM, diabetes mellitus; AG, atrophic gastritis; H. pylori, Helicobacter pylori; GLP-1, glucagon-like peptide-1; GIP, gastric inhibitory polypeptide; SIBO, small intestinal bacterial overgrowth; LPS, lipopolysaccharide. Created with BioRender.com.

### 2.1 Gastric mucosal injury mediated by hyperglycemic toxicity

Persistent hyperglycemia constitutes the core of the gastric mucosa microenvironment pathology pressure (6). Hyperglycemia directly disrupts mucosal energy metabolism and triggers a cascade of damage through multiple pathways, serving as the initial and core link.

#### 2.1.1 Oxidative stress and apoptosis

Hyperglycemia induces severe oxidative stress through multiple mechanisms, the main sources of which include mitochondrial electron transport chain overload, superoxide anion production, and NADPH oxidase activation; hyperglycemia also increases NOX-4 expression (7). In addition, AGEs induced by high glucose bind to their receptor, RAGE, thereby activating intracellular oxidative stress pathways. (See Section 4 for detailed discussion of shared ROS-NF-κB mechanisms).

Excessive ROS attacks cell membrane lipids, oxidizes functional proteins, and causes DNA fragmentation. Experiments have shown (8) that MDA and 4-HNE levels are increased, and antioxidant enzyme activity is decreased, in the gastric mucosa of diabetic animals. ROS acts as a second messenger to activate NF-κB, up-regulate pro-apoptotic Bax and down-regulate anti-apoptotic Bcl-2, resulting in Bcl-2/Bax imbalance, activation of the mitochondrial apoptotic pathway, activation of Caspase-9 and -3 cascades, and excessive cell apoptosis (9).

Super normal balance is disrupted; proliferation and apoptosis are out of balance; WeiXianTi density decreases; lamina propria thins; mucous membrane thins; this is a shrinking-core pathology. ROS also reduces the bioavailability of NO, exacerbates microcirculatory disorders, and impairs repair.

#### 2.1.2 Accumulation of AGEs and signal interference

Another key link in the long-term hyperglycemic drive in the diabetic state is the abnormal accumulation of AGEs and the excessive activation of the receptor signaling axis (10). This process and oxidative stress promote each other, and together constitute an important molecular basis for gastric mucosal injury and atrophic changes.

High glucose causes the non-enzymatic Maillard reaction to form AGEs. Gastric mucosa is rich in long-lived proteins such as collagen and laminin, which are readily covalently modified and irreversibly cross-linked by AGEs, thereby altering the matrix’s physicochemical properties and affecting cell migration and nutrient diffusion. Animal models have shown that elevated AGEs are associated with structural disorders (11). AGEs bind to RAGE, activate NF-κB and MAPK, induce the production of proinflammatory factors, and initiate chronic inflammation (12). RAGE activation also promotes ROS production by NADPH oxidase, exacerbating damage and forming a positive feedback loop (7).

AGEs-RAGE also inhibits fibroblast function, reduces collagen synthesis, and impedes healing. Cross-linking reduces mucosal compliance and barrier and increases susceptibility to acid, bile, and HP. The accumulation of AGEs is positively correlated with gland reduction and mucosal thinning, suggesting that it may inhibit proliferation and promote apoptosis (13).

#### 2.1.3 The defense and repair ability of gastric mucosa is weakened

While hyperglycemia continues to damage the gastric mucosa, diabetes also weakens its endogenous defense barrier and repair capacity through multiple mechanisms, thereby accelerating the onset and progression of atrophic gastritis.

At the same time, hyperglycemia impairs endogenous defense and repair mechanisms and accelerates atrophy. PGE_2_ is an important protective factor that increases blood flow, mucus, and bicarbonate and inhibits gastric acid (14). In the diabetic state, oxidative stress and chronic inflammation induced by hyperglycemia can inhibit cyclooxygenase (COX) activity and expression, thereby reducing PGE_2_ synthesis. Studies have shown (15) that PGE_2_ levels in the gastric mucosa of diabetic animals are significantly decreased, accompanied by impaired mucosal barrier function. The deficiency of PGE_2_ causes the gastric mucosa to lose its important “self-limiting” protection, making it difficult to heal even mild mucosal erosion, and providing conditions for the continuous progression of atrophic lesions.

Mucosal microcirculation blood flow disorder. Adequate mucosal blood flow is a prerequisite for maintaining gastric mucosal oxygen supply, nutrient delivery, and the clearance of metabolic waste products and damaging factors. Hyperglycemia inhibits endothelial nitric oxide synthase (eNOS) activity and reduces NO production (16). However, NO is the primary mediator of basal gastric mucosal blood flow and reactive hyperemia after injury. Asymmetric dimethylarginine (ADMA) is elevated in the diabetic gastric mucosa, thereby further inhibiting NO bioavailability and exacerbating mucosal microcirculatory perfusion insufficiency. Studies have confirmed (17) that gastric mucosal blood flow in diabetic animals is significantly decreased under stress, and this effect can be reversed by exogenous L-arginine. Persistent hypoperfusion causes ischemia, hypoxia, and disordered energy metabolism in the gastric mucosa, leading to a protracted defect, progressive gland loss, and mucosal atrophy.

Epidermal growth factor (EGF) and other signaling abnormalities. The repair process following gastric mucosal injury depends on a variety of growth factors that fine-regulate cell migration, proliferation, and differentiation, among which EGF and TGF-β play key roles (18). A high glucose environment can directly interfere with the production, receptor expression, and downstream signal transduction of these factors. Therefore, the process of “epithelial reconstruction” at the edge of ulcers or erosions is seriously hindered, and the integrity of the mucosa is difficult to restore. Any small damage may become the starting point of chronic atrophic lesions.

In summary, diabetes impairs the defense and repair capacity of the gastric mucosa by reducing prostaglandin synthesis, impairing mucosal microcirculation, and interfering with EGF/TGF-β signaling pathways. This vicious cycle of “weakened protection” and “sustained damage” is an important pathological basis for the high incidence and difficulty in reversing atrophic gastritis in the context of diabetes.

### 2.2 Diabetic microangiopathy and gastric mucosal ischemia

In diabetes, gastric mucosal atrophy is not caused by a single factor but by a structural degenerative change that is a co-evolution of microangiopathy driven by a long-term high-glucose environment, chronic ischemia and hypoxia, endothelial dysfunction, and failure of neurohormonal regulation.

#### 2.2.1 Microangiopathy of gastric mucosa

Diabetic microangiopathy systematically involves the gastric submucosal vascular plexus. Pathology showed that the basement membrane of gastric mucosal capillaries in diabetic patients was significantly thickened, accompanied by endothelial cell swelling, luminal stenosis or occlusion, and remodeling of microvascular structure, resulting in decreased effective perfusion density. Laser Doppler flowmetry has confirmed that the mean blood flow in each region of the gastric mucosa in patients with non-insulin-dependent diabetes mellitus (NIDDM) is significantly lower than in healthy controls and in non-diabetic patients with chronic superficial gastritis (19). Chronic hypoperfusion leads to an insufficient supply of oxygen and nutrients, obstructed clearance of metabolic waste products, and active epithelial cells, such as parietal cells and chief cells, which depend on aerobic metabolism, leading to degeneration, vacuolization, and reduction in number (20).

#### 2.2.2 Imbalance of HIF-1α signaling

Chronic gastric mucosal ischemia and a drop in tissue oxygen partial pressure can stabilize and activate hypoxia-inducible factor 1α (HIF-1α). In the long-term diabetic state, HIF-1α exhibits persistent, decompensated activation. The expression of HIF-1α mRNA and protein in the gastric mucosa of diabetic rats is significantly increased and is positively correlated with the severity of delayed gastric emptying (20). Unbalanced HIF-1α no longer plays an effective compensatory role, but transcriptionally activates various inflammatory factors and directly damages gastric mucosal epithelium, inhibiting cell regeneration and repair. At the same time, long-term hypoxia through HIF-1α-dependent or independent pathways downregulates the expression of tight junction proteins, increases mucosal permeability, makes the gastric cavity more susceptible to invasion of harmful substances, triggers and maintains chronic inflammation, and ultimately promotes atrophy.

#### 2.2.3 Endothelial dysfunction and NO system inactivation

By uncoupling eNOS, diabetes-induced oxidative stress leads to a significant decrease in NO bioavailability and an abnormal upregulation of inducible nitric oxide synthase (iNOS), which produces high concentrations of cytotoxic NO (21). The imbalance in the NO system leads to three consequences: impaired vasodilation, a blunted gastric mucosal response to the hyperemia required for physiological or post-injury repair, and aggravated ischemia. Leukocyte adhesion is associated with microthrombosis. The reduction in NO makes leukocytes more likely to adhere to the damaged endothelium, thereby releasing inflammatory mediators and blocking capillaries, forming the “no-reflux” phenomenon, while the increase in von Willebrand factor (VWF) induced by high glucose and hyperviscosity promotes microthrombosis (22). Direct mucosal protection is impaired. NO itself can promote mucus and bicarbonate secretion, and its reduction reduces mucosal tolerance to harmful stimuli.

#### 2.2.4 Failure of the neuroendocrine regulatory network

Gastric mucosal integrity depends on complex neurohumoral regulation. Diabetes mellitus is often accompanied by autonomic neuropathy, which leads to hypofunction of sensory afferent fibers. At the same time, the expression or responsiveness of ghrelin and other peptides, which have nutritional effects on gastric mucosa, is decreased, which causes the gastric mucosa to be in a state of long-term exhaustion of repair ability. Chronic ischemia caused by microangiopathy and the failure of neuroendocrine regulation overlap, and the renewal rate of the gastric mucosal epithelium cannot keep pace with the rate of injury, eventually progressing from chronic active inflammation to intrinsic gland reduction, intestinal metaplasia, and other atrophic changes.

### 2.3 Diabetic autonomic neuropathy and abnormal gastric motility

In the state of diabetes, autonomic neuropathy is the core, and microvascular disease and oxidative stress are superimposed to create a chronic pathological microenvironment that damages the integrity of the gastric mucosa (23). This environment provides a key condition for the occurrence and development of atrophic gastritis by disrupting gastric secretion, motility, and electrophysiological activity.

#### 2.3.1 Vagus nerve dysfunction and gastric acid secretion disorder

Autonomic neuropathy, particularly the decline in vagus nerve function, is the initial link in diabetes-related effects on gastric physiology. The vagus nerve is not only the core of the regulation of gastric receptivity and motility, but also the main way to stimulate gastric parietal cells to secrete gastric acid. In patients with long-term diabetes, insulin-induced hypoglycemia often shows reduced or absent gastric acid secretory response, which confirms that the vagus drive to the acid-secreting area is impaired. The loss of innervation results in markedly reduced basal and postprandial gastric acid secretion. A low-acid environment not only reduces the chemical barrier against pathogens and increases the risk of Helicobacter pylori colonization but also stimulates enterochromaffin-like cells via increased gastrin feedback, thereby inducing the long-term proliferation of neuroendocrine cells. In addition, some patients with diabetes exhibit abnormally elevated post-meal gastrin release, which may reflect a subtle imbalance in vagus nerve regulation of G cells and D cells, with the inhibitory nerve more severely damaged, leading to hypergastrinemia (24). Sustained high gastrin may be a compensatory response to low acid, but if accompanied by chronic inflammation, it may become a cofactor in inducing atrophy and metaplastic transformation of the gastric antral mucosa.

#### 2.3.2 Delayed gastric emptying and duodenogastric reflux

Diabetic gastroparesis is a typical manifestation of autonomic neuropathy involving gastroduodenal coordinated movement, and its core is a gastric antrum pylorus duodenal motility disorder. Studies have confirmed that in the diabetic state, antral contraction is weakened and frequency is dysregulated, while pyloric tension is abnormally increased, leading to a significant delay in the emptying of solid food (25). This “low antral motility and high pylorus resistance” pattern not only results in food retention, but also destroys anterograde peristalsis coordination. When intragastric pressure is elevated and the pylorus is dysfunctional, bile-acid- and pancreatic-enzyme-rich duodenal contents backflow into the stomach, causing pathological bile reflux, disrupting epithelial tight junctions, damaging cell membranes, and inducing mitochondrial dysfunction (26).

#### 2.3.3 Disturbance of gastric electrical rhythm and reduction of interstitial cells of Cajal

Diabetes impairs gastric motility at the cellular level by affecting the interstitial cells of Cajal (ICCs), the pacemaker cells of the gastrointestinal tract. Studies have shown that ICC networks in the gastric body and antrum are significantly reduced and morphologically abnormal in patients with diabetic gastroparesis and in animal models (27). ICC loss directly leads to abnormal origin and conduction of the slow-wave rhythm, and the stomach fails to produce protraction contraction, resulting in ineffective, fragmented contractions. ICC reduction is caused by multiple factors, with oxidative stress as the core driver of hyperglucose-induced mitochondrial dysfunction and ROS overproduction, and inducing ICC apoptosis via the MAPK/NF-κB pathway (28). Microangiopathy causes ischemia and hypoxia in the gastric mucosa, thereby exacerbating the energy crisis in ICC. At the same time, there is a decrease in protective M2 macrophages and an increase in pro-inflammatory M1 macrophages, which secrete TNF-α to directly damage ICC (29). The imbalance between autophagy and apoptosis in a high-glucose environment also accelerates the demise of ICC (30).

In conclusion, diabetes is not an independent cause of atrophic gastritis directly, but constructs a highly proinflammatory gastric environment through the pathological chain of autonomic neuropathy, motility disorder, and mucosal injury (31). Vagus neuropathy weakens the acid barrier and disrupts gastrointestinal hormonal regulation. Gastroparesis with bile reflux results in a prolonged chemical attack. Disruption of the ICC network causes the stomach to lose its ability to clean itself and empty. These three factors exacerbate one another, forming a vicious circle of “delayed emptying - bile reflux - mucosal injury -ICC loss - motility deterioration”. In the microenvironment of low acidity, bile-rich conditions, ischemia, and high oxidative stress, the gastric mucosa is repeatedly damaged and repaired, eventually leading to gland atrophy, intestinal metaplasia, and even precancerous lesions.

### 2.4 Increased susceptibility to Helicobacter pylori infection and worse outcome after infection

There is a complex two-way interaction between diabetes mellitus and Helicobacter pylori infection that ultimately accelerates the malignant transformation of the gastric mucosa, progressing from chronic inflammation to atrophic gastritis and even intestinal metaplasia. Metabolic abnormalities in diabetes mellitus increase the host’s susceptibility to HP infection, thereby significantly accelerating the clinical course of HP-associated gastritis.

#### 2.4.1 The molecular basis of high glucose microenvironment promoting Helicobacter pylori colonization and adhesion

There is a series of pathological changes in the gastric mucosal microenvironment of diabetic patients that promote Hp colonization. Firstly, long-term hyperglycemia leads to abnormal glycosylation patterns of mucin in the mucous layer of the gastric mucosa. Specifically, under hyperglycemia, the accumulation of non-enzymatic glycosylation end products (AGEs) of mucin O-linked glycan chains may expose additional glycosyl receptor structures that bind specifically to Hp adhesins, thereby enhancing the efficiency of molecular recognition and adhesion between bacteria and gastric epithelial cells.

Secondly, the inhibition of gastric acid secretion by hyperglycemia constitutes the second layer of the mechanism promoting colonization. Although the effect of diabetes on gastric acid secretion is different in stages, which may lead to high gastric acid in the early stage and low gastric acid in the late stage of gastric neuropathy, long-term hyperglycemia eventually leads to the impairment of gastric acid barrier function by damaging the function of the vagus nerve and gastric parietal cells. Hydrochloric acid in gastric juice is the first line of defense against oral ingestion of H. pylori; gastric acidity is needed to significantly reduce the infection threshold. Related study (32): Hp fasting blood glucose levels in serum were significantly higher than those in the serum-negative group, indicating hyperglycemia, and there was a positive correlation between Hp infection and the link, excluding other metabolic factors such as obesity, which still exists. Follow-up studies further reveal that high blood sugar can be mitigated by gastric mucosal barrier function, thereby promoting long-term engraftment of Hp (33).

#### 2.4.2 The vicious cycle of immune bias and persistent H. pylori infection in the diabetic state

Patients with diabetes present a series of systemic defects in innate and adaptive immune functions, which directly lead to the inability to be effectively eliminated after Hp infection, resulting in a persistent chronic infection state.

At the level of innate immunity, related studies have confirmed that the chemotaxis, phagocytosis, and intracellular killing functions of neutrophils are significantly impaired in diabetic patients. Its dysfunction makes it difficult to eliminate Hp in the early stage of infection. At the same time, the function of macrophages in the diabetic state is skewed toward a pro-inflammatory phenotype, laying the groundwork for subsequent atrophy and metaplasia (34).

At the level of adaptive immunity, there are complex interactions between the autoimmune background of diabetes and Hp infection. Relevant studies have found significant co-aggregation of PCA positivity with anti-GAD and anti-thyroid peroxidase antibodies in patients with diabetes, suggesting a systemic autoimmune tendency (35). HP infection may act as a trigger for or amplify this autoimmune response through molecular mimicry (36).

HP induces the massive production and secretion of the potent immunosuppressive cytokine IL-37 by activating TLR2, TLR4, and NOD1 receptors in gastric epithelial cells. By inhibiting TLR-mediated NF-κB activation and IL-8 production, IL-37 prevents the formation of immune synapses of T cells and B cells, inhibits the chemotaxis and migration of T cells, and inhibits the proliferation of T cells and B cells, thereby systematically inhibiting the host immune response. In the diabetic state, the high glucose environment itself has activated chronic inflammation and induced immune tolerance through the AGEs-RAGE axis. HP further superimposed the immunosuppressive effect through multiple pathways, forming a vicious cycle of “persistent infection - failure of immune clearance - chronic inflammation - accumulation of tissue damage - atrophy and metaplasia”.

#### 2.4.3 Enhancement of the Helicobacter pylori-induced atrophy pathway

Atrophic gastritis is a pathological process in which the parietal and chief cells of the gastric mucosa are progressively lost in response to long-term chronic inflammatory stimulation, and the glandular structure is destroyed and replaced by metaplastic epithelium.

The hyperglycemic state inherent in diabetes leads to dysfunction of the mitochondrial electron transport chain and a continuous increase in ROS production. Hp infection, especially the virulent strains carrying cagPAI, delivers CagA proteins into host epithelial cells through its type IV secretion system, which further activates the NADPH oxidase system and exacerbates oxidative stress levels. In the gastric mucosa, the same level of oxidative stress can lead to cumulative damage to parietal cells’ mitochondrial DNA, exceeding the compensatory capacity of stem cells’ regenerative ability, resulting in irreversible gland atrophy.

High levels of proinflammatory cytokines already exist in the circulation of patients with diabetes. On this basis, Hp infection further activates the local NF-κB signaling pathway in the gastric mucosa, induces epithelial cells to produce IL-8, and recruits neutrophils to the site of infection. In a study of 105 patients with histologically confirmed atrophic gastritis of the gastric body (37), a significantly higher proportion of anti-intrinsic factor antibody (IFA) positive patients had coexisting autoimmune diseases than IFA-negative patients. Gastric body atrophy associated with Hp infection reduces gastric acid secretion, thereby reducing negative feedback on G cells and causing hypergastrinemia. Gastrin not only stimulates the proliferation of enterochromaffin-like (ECL) cells but also may accelerate the terminal differentiation and senescence of parietal cells through mechanisms that have not yet been fully elucidated. In diabetic patients, hyperglycemia itself impairs the function of G and D cells, which may further strengthen this pathological link.

The abnormal activation of TGF-β, Wnt, and Notch signaling pathways in the chronic inflammatory microenvironment can induce EMT in gastric epithelial cells, an important initial step in intestinal metaplasia. Diabetes-related metabolic abnormalities may synergize with inflammatory signals generated by Hp infection, via activation of the PI3K/Akt signaling pathway, to promote the spread and consolidation of metaplastic changes (38).

In conclusion, diabetes promotes the development and progression of HP-associated atrophic gastritis through the following three core pathways. One is that the high glucose microenvironment enhances the density and persistence of H. pylori colonization by altering the mucin glycosylation pattern and weakening the gastric acid barrier. The second is the deficiency in innate and adaptive immunity associated with diabetes mellitus, combined with HP-induced IL-37-mediated immunosuppression, resulting in an inability to effectively eliminate bacteria and in chronic, persistent infection. The third is the inherent oxidative stress, chronic inflammatory state, and autoimmune tendency of diabetes, which synergistically act with Hp virulence factors to amplify DNA damage in gastric epithelial cells and accelerate the progress of the “Correa cascade”. Active screening and eradication of Hp infection is not only necessary to control gastrointestinal symptoms, but also an important strategy to prevent atrophic gastritis and its subsequent risk of gastric cancer.

### 2.5 Imbalance of gastrointestinal hormone network

Long-term hyperglycemia, autonomic neuropathy, and disturbances in local microcirculation not only impair islet β-cell function but also cause significant structural and functional remodeling of endocrine cells widely distributed throughout the gastrointestinal mucosa.

#### 2.5.1 Paradoxical effects of hypergastrinemia

Hypergastrinemia is the core pathological link linking diabetes and atrophic gastritis (39). The mechanism of its production has a dual basis. The first is the direct loss of negative feedback from gastric acid; in the diabetic state, reduced gastric acid secretion underlies hypergastrinemia (40). Physiologically, the hyperacidic environment in the gastric lumen inhibits gastrin secretion by G cells by stimulating somatostatin release from antral D cells. When diabetes leads to chronic gastric acid deficiency, the strong negative feedback inhibition is relieved, and G cells enter a “disinhibitory” state that compensates for the persistent secretion of large amounts of gastrin.

Secondly, the pro-atrophy and pro-tumor effects of high gastrin are contradictory. At physiological concentrations, gastrin is an important trophic hormone for gastric mucosa, especially for parietal cells. However, under prolonged, extreme hypergastrinemia induced by diabetes, the effects are reversed. High gastrin persistently stimulates enterochromaffin-like cells located in the fundic glands, leading to their hyperplasia and progression to dysplasia. More critically, long-term high levels of gastrin may induce further functional decline or apoptosis of parietal cells while promoting ECL cell proliferation through unknown or non-classical pathways, accelerating the evolution of the fundic gland region from inflammation to atrophy (41).

#### 2.5.2 Attenuation of the inhibitory effect of somatostatin

Somatostatin, secreted by gastric mucosal D cells, is the core inhibitory signal regulating gastric acid and gastrin. In diabetic gastrointestinal lesions, structural and functional defects in D cells are key to the imbalance in the hormone network. Related studies have shown that diabetes may lead to a decrease in the number of D cells in the gastrointestinal tract or the depletion of secretory granules in D cells, which reduces the ability of D cells to secrete SST and thus cannot effectively respond to the signal of reduced gastric acid and enhance SST release (42).

Under normal physiological conditions, gastric acid secretion is subject to sophisticated negative feedback regulation. SST is released by D cells in the antrum of the stomach in response to a decrease in gastric acid levels, and SST inhibits gastrin secretion by G cells. As gastrin decreases, the stimulating effect on gastric acid secretion diminishes, eventually restoring the balance of gastric acid levels. However, in the case of diabetes combined with atrophic gastritis, this negative feedback loop is disrupted due to inhibition failure, and gastric acid depletion should activate D cells to secrete SST, but due to impaired D cell function and insufficient secretion of SST, its inhibitory effect on G cells is weakened or even disappears (43). As a result, G cells enter a disinhibitory state and oversecrete gastrin, resulting in abnormally elevated blood gastrin levels. High gastrin levels continuously stimulate ECL cells and induce apoptosis of gastric parietal cells, thereby further aggravating gastric mucosal atrophy and eventually leading to severe, persistent low gastric acid levels, forming a vicious cycle. This forms a malignant positive feedback loop of “G-D cell” imbalance (44). The diminished inhibitory effect of SST, which not only directly disinhibits G cells but may also relieve local inhibition of inflammatory responses, further exacerbates the disordered immune microenvironment of the gastric mucosa (45).

#### 2.5.3 Abnormal ghrelin and motilin

In addition to the core axis of gastrin-SST, abnormalities in other gastrointestinal hormones also indirectly promote the occurrence and progression of atrophy. Ghrelin is mainly secreted by X/A-like cells in the gastric fundus and has strong appetite-promoting and gastrointestinal motility effects. Motilin, in turn, is mainly responsible for initiating the transitional motor complex during digestion and scavenging gastrointestinal contents (46).

Reduced Ghrelin and motilin levels, along with dysrhythmia, directly contribute to gastric motility disorders and abnormal feeding feedback (47). This physical motility disorder, on the one hand, leads to retention of gastric contents and bacterial overgrowth, and the metabolites of these bacteria may produce chronic inflammatory stimulation of the gastric mucosa. On the other hand, motility disorders interfere with the normal physical self-cleaning and repair rhythm of the gastric mucosa, making it more difficult for the damaged mucosa to recover from continuous immune and chemical attacks in the absence of sufficient blood flow and nutrient supply, thereby indirectly promoting the pathological evolution of gastric mucosa from inflammation to atrophy (48).

To sum up, diabetes is not simply the cause of atrophic gastritis, but also high blood sugar, complex factors such as autonomic neuropathy, and deeply disturbed gastrointestinal hormones, which disrupt the steady state of the network. Hypergastrinemia, decreased somatostatin inhibition, and abnormal ghrelin and motilin levels are intertwined and collectively constitute a systemic, networked pathological mechanism that promotes the occurrence and progression of atrophic gastritis in the diabetic state.

## 3 Reverse effects of atrophic gastritis on glucose metabolism and progression of diabetes mellitus

Atrophic gastritis (AG) is characterized by the reduction of intrinsic glands in the gastric mucosa. The core pathology of AG is progressive destruction of gastric parietal cells, resulting in a significant decrease in gastric acid secretion and severe deficiency of intrinsic factor (IF). It is important to distinguish between established causal relationships and associative findings in this section: while nutrient malabsorption (B_12_, iron) and hormonal dysregulation have been mechanistically linked to β-cell dysfunction and insulin resistance in preclinical studies, large-scale prospective clinical evidence confirming these as direct causal pathways in humans remains limited. Most human data to date are derived from cross-sectional or retrospective studies demonstrating associations, and interventional trials showing that correcting AG-related deficiencies directly improves glycemic outcomes are scarce. Through disruption of the nutrient absorption barrier caused by gastric mucosal lesions, AG exerts a profound “reverse effect” on glucose homeostasis and diabetes progression by interfering with key nutrient metabolism.

### 3.1 Decreased gastric acid secretion and nutrient malabsorption

The loss of the number and function of gastric parietal cells fundamentally destroys the “digestion and absorption” function of the stomach. Gastric acid deficiency directly hinders the release of conjugated vitamin B_12_ from food, and intrinsic factor is an absolute prerequisite for B_12_ to be specifically absorbed in the terminal ileum (49). As a result, AG patients are often in a progressive, occult state of vitamin B_12_ deficiency (50). At the same time, gastric acid can reduce dietary Fe^3+^ to soluble Fe^2+^, thereby severely impairing iron bioavailability. Together with the possible hidden blood loss in atrophic gastritis, iron deficiency anemia is another common complication.

#### 3.1.1 Vitamin B_12_ deficiency and hyperhomocysteinemia

Ag-induced B_12_ deficiency first disturbs one-carbon metabolism. B_12_ is a methionine synthase coenzyme that catalyzes the methylation of homocysteine (Hcy) to methionine (51). This reaction is blocked in B_12_ deficiency, triggering hyperhomocysteinemia (HHcy) (52). Multiple studies have established HHcy as an independent risk factor for insulin resistance and type 2 diabetes mellitus (T2DM) (53). The mechanism of HHcy involves oxidative stress, endothelial damage, and β-cell damage. Oxidation to produce a large number of reactive oxygen species, HHcy itself directly damages the vascular endothelial cells, reduces the NO bioavailability, which not only increases the risk of diabetes vascular and microvascular complications, but also damages skeletal muscle and adipose tissue blood perfusion and signal transduction, and compounds peripheral insulin resistance (54). At the same time, high Hcy levels can induce apoptosis of pancreatic β-cells through endoplasmic reticulum stress and mitochondrial dysfunction pathways, reduce the number of functional β-cells, and impair the ability to secrete to compensate for insulin resistance (55). Of note, a significant inverse association between B_12_ deficiency and lower HbA1c levels in T2DM patients with long-term metformin use may reflect the accumulation of higher drug doses in those with good glycemic control, and metformin is known to further interfere with B_12_ absorption (56), revealing an additive effect of the drug and the primary disease on B_12_ depletion (57).

#### 3.1.2 Iron deficiency and glucose metabolism

Iron is a prosthetic group for a variety of enzymes key to energy metabolism. AG-induced iron deficiency poses a direct challenge to energy-intensive insulin-sensitive tissues. The mechanism mainly involves impaired mitochondrial function and the secretion of a function-damaging factor by beta cells. Iron deficiency limited the activity of the mitochondrial electron transport chain, particularly the function of complex IV (cytochrome c oxidase), resulting in reduced Krebs cycle activity and reduced phosphate coupling, thereby decreasing ATP production efficiency (58). Encouraged skeletal muscle and liver cells in glycolytic energy, increased intracellular lipid accumulation, causing fat toxicity, activating protein kinase C (PKC) pathway, such as directly inhibiting phosphorylation of insulin receptor substrate (IRS), inducing insulin resistance (59). In addition, β-cells rely on sufficient ATP to trigger glucose-stimulated insulin secretion (GSIS), and mitochondrial dysfunction caused by iron deficiency directly impairs β-cell energy metabolism and reduces their response to elevated blood glucose (60). Chronic iron deficiency may also lead to tissue hypoxia, which further affects β-cell identity and function through the hypoxia-inducible factor (HIF) pathway.

In summary, atrophic gastritis constitutes a bi-axis “metabolic trap” centered on vitamin B_12_-homocysteine and iron metabolism through gastric acid and intrinsic factor deficiency. Rather than directly attacking islets by autoimmunity or inflammation, atrophic gastritis indirectly and progressively worsens insulin resistance and impairs β-cell function and survival by depriving islets of essential micronutrients for glucose metabolism.

### 3.2 Gastrointestinal hormone feedback adjustment disorder

Atrophic gastritis not only impairs digestive function through atrophy of the gastric mucosa but also exerts a complex, bidirectional “reverse effect” on the homeostasis of glucose metabolism by reconstructing the gastric hormone network (61). The traditional view is that hypergastrinemia may improve glucose metabolism (62). However, in the pathological context of atrophic gastritis, extreme, non-physiological dysregulation of two key hormones, gastrin and ghrelin, may instead exacerbate insulin resistance, impair β-cell function, and be closely associated with the progression and complications of specific types of diabetes (63).

#### 3.2.1 Regulation of insulin secretion by extreme hypergastrinemia

Antral G cells show persistent activation due to loss of negative feedback inhibition by gastric acid, resulting in abnormally elevated circulating gastrin (64). Although mild-to-moderate hypergastrin is thought to promote β-cell proliferation and insulin-sensitizing effects, its effects may be reversed under the extreme pathological conditions of atrophic gastritis, which manifests as negative regulation of glucose metabolism. One of the core mechanisms is the abnormal activation of CCK-B receptors in beta cells. Cholecystokinin B receptor (CCK-BR) is also expressed on islet β-cells (65).

Under physiological conditions, pulsatile gastrin release after a meal can enhance glucose-stimulated insulin secretion (GSIS), but in chronic hypergastrinemia, long-term exposure of β-cells to superphysiological concentrations of gastrin can desensitize and internalize CCK-BR, impairing their ability to respond normally to a glucose load (66). Excessive gastrin signaling overactivates PLC, IP3, and MAPK pathways. Although prolonged strong signal stimulation can initially promote insulin secretion, it may eventually deplete insulin reserves, accelerating the transition of β-cells from compensatory to decompensated states (67). In addition, persistently high gastrin levels may also interfere with the expression and nuclear translocation of β-cell-specific transcription factors, or even induce dedifferentiation or transdifferentiation of β-cells into other endocrine cell types, thereby irreversibly reducing functional β-cell mass and aggravating impaired glucose tolerance (68). It is important to note that such hypergastrinemia may be asymptomatic, masking its pancreatic hormonal effects. Conversely, hypergastrin may interfere with normal signaling integration of the gut-islet axis, antagonizing GLP-1 or GIP signaling, resulting in diminished overall incretin effects and making glycemic fluctuations more difficult to fine-tune by diet and endogenous insulin (69).

#### 3.2.2 Persistent alterations in Ghrelin secretion patterns

Ghrelin is mainly secreted by X/a-like cells of the gastric fundus mucosa and is the only known potent orexigenic peripheral hormone (70). Atrophic gastritis, especially when involving the body and fundus of the stomach, directly destroys the main source of ghrelin secretion, resulting in a fundamental change in its secretion pattern (71).

Physiologically, circulating ghrelin increases before a meal to initiate appetite and decreases rapidly after a meal. This “hunger signal” is essential for maintaining normal energy intake. Atrophic gastritis leads to an absolute reduction in the number of X/A-like cells and the abnormal formation of a ghrelin peak before meals, which may lead to early satiety and loss of appetite. In the long term, atrophic gastritis may aggravate nutritional imbalance and metabolic vulnerability in diabetic patients. In the pancreas, ghrelin acts on β-cells in an autocrine or paracrine manner to inhibit GSIS via the Gαi pathway, thereby preventing excessive basal insulin release. In the absence of ghrelin, the physiological suppression is lifted, which may lead β-cells to secrete insulin even at inappropriately low glucose levels, perturb glucose homeostasis, or disrupt the insulin secretion rhythm and β-cell stress in the long term (72).

In addition, ghrelin can promote skeletal muscle synthesis, exert anti-inflammatory effects, and protect mitochondrial function. Exogenous ghrelin improved mitochondrial oxidative capacity and AKT phosphorylation in a muscle atrophy model. Chronic ghrelin deficiency associated with atrophic gastritis may impair these muscle protective mechanisms, potentially linking the pathophysiology of “diabetic sarcopenia” common in diabetic patients, which in turn is closely associated with increased insulin resistance and an increased risk of falls (73).

Taken together, atrophic gastritis exerts profound negative effects on islets through remodeling of the gastric endocrine axis. Extreme hypergastrinemia increases the risk of β-cell depletion and dedifferentiation through CCK-BR sustained hyperstimulation, while significant ghrelin deficiency, abrogating the fine inhibition of insulin secretion, deprives physiological starvation-initiating signals and may impair the metabolic and functional reserve of muscle tissue.

### 3.3 Systemic metabolic effects of gut dysbiosis

Atrophic gastritis progresses, with severely insufficient or absent gastric acid production due to progressive loss of gastric body wall cells and key microbial defenses in the small intestine (74). It causes SIBO, changes the number and composition of the flora, and has a profound negative impact on systemic glucose metabolism through the “liver-gut-pancreas” axis (75).

#### 3.3.1 Low stomach acid induced SIBO

Gastric acid is the first chemical barrier, which inactivates foreign microorganisms and maintains the sterile stomach and proximal small intestine. Atrophic gastritis causes parietal cell depletion and reduces gastric acid to low or absent levels, allowing oral, nasopharyngeal, and upper gastrointestinal microorganisms to survive and migrate to the small intestine, bypassing defense barriers (76).

After the barrier is damaged, the intestinal flora reaches the SIBO threshold; facultative anaerobes and anaerobes reverse-migrate to the proximal segment, and the proportion of beneficial bacteria, such as Lactobacillus, decreases, resulting in a dual imbalance in both quantity and quality that may affect distal metabolism (77). Clinically, SIBO causes abdominal distension, diarrhea, steatorrhea, and, in diabetic patients, irregular digestion and absorption, marked fluctuations in blood glucose, and the appearance of “fragile blood glucose”, which interferes with management (78).

#### 3.3.2 The liver-gut-pancreas axis effect of microbial metabolites and systemic insulin resistance

SIBO-induced dysbiosis alters metabolite profiles, particularly LPS and SCFA. LPS is an outer membrane component of Gram-negative bacteria. Intestinal LPS load increases during overgrowth. Abnormal bacterial flora disrupts tight junctions and increases permeability (79). LPS passes through the portal vein via paracellular transport, resulting in a persistent low-grade “metabolic endotoxemia”. LPS activates Kupffer cells and macrophages via TLR4, triggering TNF-alpha and IL-6; the JNK and IKK/NF-κB pathways predominantly interfere with insulin signaling, inducing resistance (80). After entering the systemic circulation, it amplifies systemic inflammation and causes the resistance to evolve from local to systemic. Physiologically, SCFAs improve insulin sensitivity, enhance barrier function, and have anti-inflammatory effects through GPR41/GPR43 and by inhibiting histone deacetylases (81). But when SIBO ferments in the small intestine, resulting in SCFA spectral change, and propionic acid, butyric acid, and acetic acid excess lipid synthesis (82). SCFA promotes GLP-1 secretion via FFAR2/FFAR3, whereas SIBO abnormalities blunt the L-cell response and impair the postprandial GLP-1 elevation (83).

#### 3.3.3 Effects on incretin (GLP-1/GIP) axis

Dysbiosis impairs the incretin axis in multiple ways. The microbiota is involved in the enterohepatic circulation and transformation of bile acids. SIBO changes the enzyme activity and remodels the bile acid pool. Bile acids are ligands for L cell TGR5 and FXR (84). Activation of TGR5 promotes GLP-1 secretion, whereas excessive activation of FXR inhibits it. Dysbiosis leads to changes in the bile acid profile, breaking the balance, allowing inhibition to prevail, and impairing GLP-1 release (85).

In addition, abnormal metabolites and inflammatory mediators directly act on L cells, such as LPS activating TLR4, triggering local inflammation and interfering with GLP-1 synthesis. Microbial changes in nutrient metabolism affect L-cell sensing, resulting in abnormal GLP-1 and GIP secretion phase and amplitude after eating. Clinically, the incretin effect is diminished, insulin secretion is blunted, and postprandial blood glucose levels worsen (86).

In conclusion, atrophic gastritis may adversely affect glucose homeostasis through two principal pathways (1): “reduced gastric acid - SIBO - altered metabolites - LPS/TLR4-mediated metabolic endotoxemia - systemic insulin resistance”; and (2) “dysbiosis - altered SCFA profile/bile acid metabolism - L-cell dysfunction - impaired GLP-1/GIP secretion - deteriorated postprandial glucose regulation.” Through a shared inflammatory signaling network, endotoxin-induced systemic low-grade inflammation and mutual reinforcement of these pathways damage L-cell function. Reduced GLP-1 further weakens the anti-inflammatory and protective effects of insulin, creating a vicious cycle.

## 4 The common soil and mechanism of the coexistence of the two diseases overlap

Diabetes and atrophic gastritis share a “common soil” of chronic low-grade inflammation, autoimmune dysregulation, and oxidative stress. Diabetes promotes atrophy of the gastric mucosa, and atrophic gastritis impairs glucose metabolism through gastric acid deficiency, dysbiosis, and nutritional deficiencies, which are mutually causal. The shared mechanisms constituting this “common soil” are summarized in **Figure 2**.

**Figure 2:**
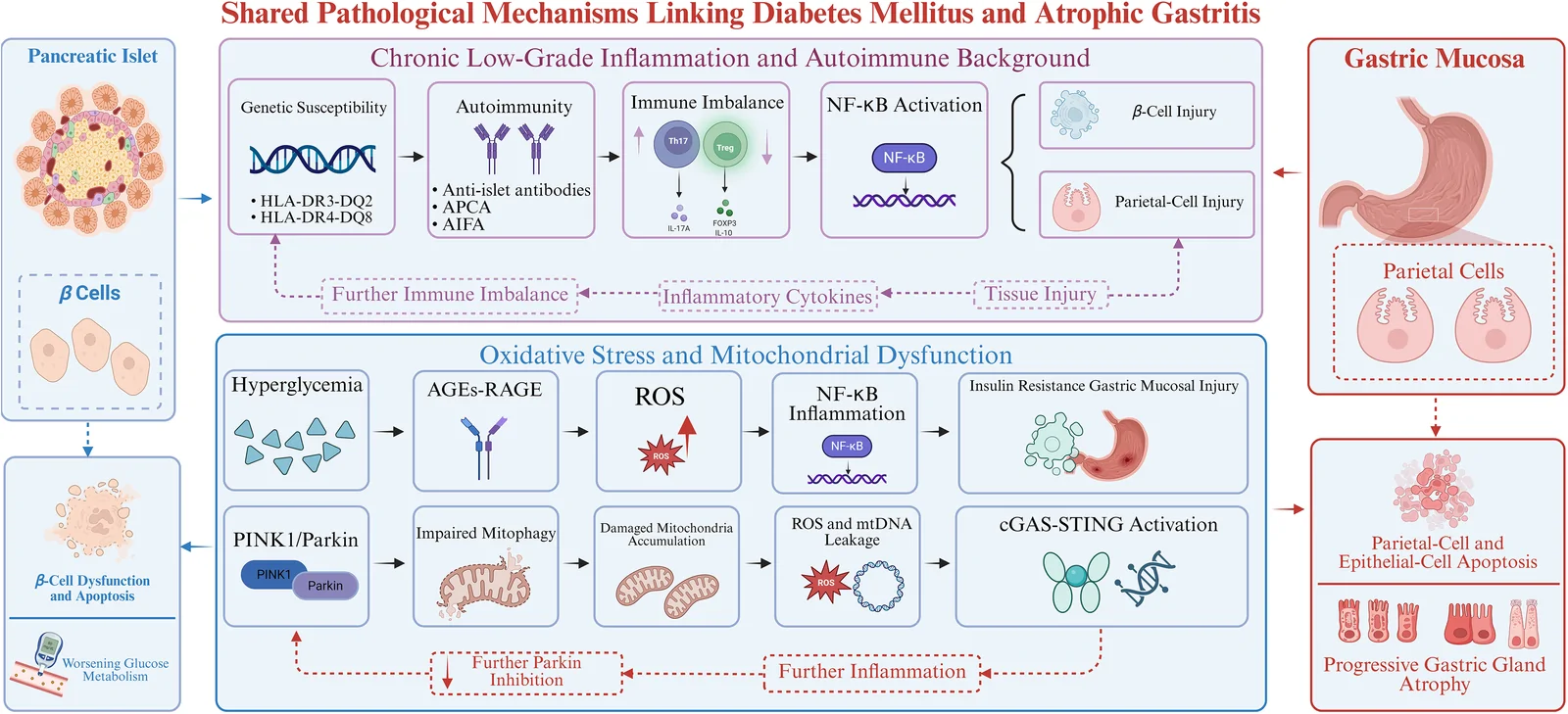
Shared pathological mechanisms linking diabetes mellitus and atrophic gastritis. Both diseases share a background of genetic susceptibility (HLA class II), autoimmunity, and chronic low-grade inflammation, in which Th17/Treg imbalance and sustained NF-κB activation may contribute to concurrent injury of pancreatic β-cells and gastric parietal cells. Hyperglycemia, AGEs-RAGE signaling, and impaired PINK1/Parkin-mediated mitophagy further promote ROS accumulation, mitochondrial DNA leakage, and cGAS-STING activation, establishing a feed-forward cycle of inflammation and cellular injury that may drive β-cell dysfunction and progressive gastric gland atrophy. Solid arrows indicate established pathways; dashed arrows indicate proposed links. AGEs, advanced glycation end products; RAGE, receptor for AGEs; ROS, reactive oxygen species; NF-κB, nuclear factor-κB; Th17, T helper 17; Treg, regulatory T cell; PINK1, PTEN-induced kinase 1; cGAS, cyclic GMP-AMP synthase; STING, stimulator of interferon genes. Created with BioRender.com.

### 4.1 Chronic low-grade inflammation and autoimmune background

The comorbidity of diabetes and atrophic gastritis is difficult to explain by the damage of a single organ. In fact, they share a set of pathological “common soil” with chronic low-grade inflammation and autoimmune disorders as the core. Comorbidity results from shared genetic susceptibility genes (87) and autoimmune abnormalities, and the increased positive rate of APCA/AIFA in diabetic patients suggests an independent role of autoimmunity (88). The Th17/Treg imbalance is the key. Th17 proinflammatory destroys islet β-cells and gastric parietal cells, and Treg deficiency impairs tolerance. Hyperglycemia activates the AGEs-RAGE axis, breaking the immune immunity of gastric mucosa and inducing autoimmune gastritis (89).

#### 4.1.1 Shared susceptibility genes and autoantibodies

Both diseases have a clear genetic basis, with HLA class II haplotypes as the primary focus. Genome-wide studies have confirmed HLA-DRB103 (DR3-DQ2) and 04 (DR4-DQ8) as common risk factors, with DR3-DQ2 homozygotes having the highest risk (90). Anti-islet antibodies and APCA/AIFA can be detected in diabetic patients, with a positive rate of about 9-10%. Positive GADA is positively correlated with AIG risk. Hyperglycemic chronic inflammation can break immune tolerance through molecular mimicry (91).

#### 4.1.2 Common roles of Th17/Treg cell imbalance

Th17/Treg imbalance was observed in both diseases. Th17 secretion of IL-17A activates NF-κB and destroys target cells, while Treg, due to FOXP3/IL-10 decline and insufficient inhibition. STAT3 over-activation drives imbalance, promotes RORγt-mediated Th17 differentiation, and inhibits FOXP3. Animal experiments have confirmed that STAT3 inhibition can reduce Th17 infiltration, increase Treg cells, and alleviate atrophy. Hyperglycemia damages the gastric mucosa through AGEs-RAGE signaling; autonomic neuropathy causes bile reflux; metformin interferes with B_12_ absorption; and superimposed intrinsic factor deficiency creates a vicious cycle that accelerates atrophy progression (92).

In the reverse effect of atrophic gastritis on the progression to diabetes, gastric acid deficiency promotes the proliferation of opportunistic pathogens, and LPS activates TLR4, inducing insulin resistance. Dysbacteriosis alters the SCFA spectrum and reduces GLP-1 secretion. Hypergastrinemia may affect islet β-cells. B_12_ or iron deficiency elevates methylmalonic acid and homocysteine, which are associated with insulin resistance and neuropathy. Disturbed gastric emptying leads to postprandial hypoglycemia and interferes with glucose-lowering management.

In summary, diabetes and atrophic gastritis constitute a bidirectional, mutually reinforcing pathological network at the genetic, immune, metabolic, and nutritional levels. This finding suggests that screening for APCA, AIFA, and gastric mucosal atrophy status should be routinely performed in patients with diabetes, and glucose metabolism and islet autoantibodies should also be evaluated in patients with confirmed atrophic gastritis to achieve early intervention and comorbidity management.

### 4.2 Common pathways of oxidative stress and mitochondrial dysfunction

Building on the mechanisms described in Sections 2 and 3, this section synthesizes the shared pathways that constitute the common soil. The pathological basis of the comorbidity of diabetes and atrophic gastritis is the oxidative stress overload induced by the hyperglycemic microenvironment and local chronic inflammation of the gastric mucosa, and the cascade failure of the mitochondrial quality control system. Mitochondria, as the hub of energy metabolism and REDOX signaling, show similar damage patterns in the two diseases, and through the positive feedback amplification of the ROS-NF-κB inflammatory axis and the cell fate determination of the coupling of autophagy and apoptosis, the systemic metabolic disorders and gastric mucosal degeneration are closely coupled to form a coexisting core pathological network.

#### 4.2.1 Synergistic activation of ROS NF-κB axis

Hyperglycemia-driven mitochondrial ROS burst and its direct proinflammatory effect. Chronic hyperglycemia enhances electron leakage from ETC complexes I and III and increases rates of superoxide anion and hydrogen peroxide production (93). At the same time, AGEs activate NADPH oxidase upon binding to RAGE, forming a double oxidative burden (94). Excess ROS both oxidizes biological macromolecules and acts as second messengers to initiate NF-κB inflammatory signaling (95). At the molecular level, ROS induces IκBα phosphorylation and degradation, which activates NF-κB translocation into the nucleus, and causes mtDNA leakage to the cytoplasm, activating cGAS, which in turn activates STING, thereby enhancing the transcriptional activity of NF-κB (96).

Local inflammation of the gastric mucosa: infiltrating immune cells continue to release TNF-α, IL-1β, and other factors, maintaining high phosphorylation of NF-κB, which in turn upregulates the transcription of these factors, thereby constituting a local positive feedback loop (97). When the two diseases coexist, bidirectional cross-amplification forms a systemic cycle of “hyperglycemia - mitochondrial ROS - NF-κB - circulating inflammatory factors - insulin resistance - hyperglycemia - aggravated gastric mucosal injury.” Even if the initial factors are controlled, the activated NF-κB signal can still maintain tissue inflammation and drive disease progression (98).

#### 4.2.2 Mitophagy disorders and cell death

Regulation mechanisms of the PINK1/Parkin pathway in physiological and pathological conditions. The PINK1/Parkin pathway is the most intensively studied mechanism of selective mitophagy (99). In the PINK1/Parkin pathway, PINK1 is rapidly degraded in healthy mitochondria. PINK1 accumulates in the outer membrane upon loss of membrane potential, phosphorylates ubiquitin and aggregates Parkin, ubiquitinates the outer mitochondrial membrane, is recognized by the autophagy receptor, and undergoes LC3-mediated clearance (100).

In diabetes and atrophic gastritis, the expression of PINK1 and Parkin is significantly down-regulated, accompanied by mitochondrial accumulation and blocked autophagic flux. Mechanistically, high-glucose oxidative stress inhibits Parkin transcription through FOXO1, and TNF-α activates the NF-κB binding negative element of the promoter to further suppress transcription (101). Meanwhile, nitrosylation of Parkin by reactive nitrogen species reduces Parkin activity, thereby synergistically disrupting autophagy. After the accumulation of damaged mitochondria, the mPTP opens, releasing cytochrome c and AIF and activating the caspase cascade to initiate apoptosis. Bax/Bak oligomerization amplifies the signal, leading to mtDNA leakage via cGAS/STING, thereby enhancing caspase-8 activation and culminating in synchronous apoptosis of gastric mucosal epithelium and pancreatic β-cells. The former exacerbates histological progression, while the latter worsens glycemic control (102).

Impaired autophagy is tightly coupled to NF-κB activation. Impaired autophagy leads to mtDNA leakage, activating STING, which collaborates with p65 to enhance the expression of inflammatory factors, thereby promoting the production of inflammatory products that inhibit Parkin and increase ROS, further inhibiting autophagy. The positive feedback loop of “impaired autophagy -mtDNA leak -STING/NF-κB activation - inflammatory factor -Parkin inhibition - further impaired autophagy” becomes the key to the difficulty of pathological reversal (103).

To sum up, diabetes and comorbidity of atrophic gastritis by chronic low-grade inflammation, autoimmune disorders, oxidative stress, and mitochondrial dysfunction as a general soil driver. A systemic injury network of continuous activation is formed by the synergistic cross-amplification of reciprocal mechanisms, which simultaneously promotes the synchronous functional failure of gastric mucosal epithelium and islet β-cells.

Key nodal points connecting the bidirectional interaction. Several molecular hubs serve as critical interfaces that directly link the DM→AG and AG→DM arms of the cycle. First, the ROS-NF-κB axis functions as a shared inflammatory amplifier: hyperglycemia-driven ROS activates NF-κB in gastric mucosa (DM→AG), while AG-induced metabolic endotoxemia and nutrient deficiency further sustain NF-κB activation systemically, worsening insulin resistance (AG→DM). Second, the PINK1/Parkin mitophagy pathway represents a common point of mitochondrial quality control failure: high glucose suppresses Parkin in gastric epithelial cells, and AG-induced B_12_/iron deficiency impairs mitochondrial function in β-cells, with both converging on defective mitophagy. Third, Th17/Treg imbalance provides a unifying immune mechanism that simultaneously drives autoimmune attack on parietal cells and pancreatic β-cells. Fourth, the gastrin-ghrelin hormonal axis acts as a bidirectional metabolic linker: diabetes-induced autonomic neuropathy alters gastrin and ghrelin secretion to promote gastric atrophy, while AG-induced destruction of fundic X/A cells further disrupts ghrelin signaling to worsen glucose homeostasis. Despite these conceptual connections, direct evidence from simultaneous biomarker profiling in dual-disease cohorts and interventional studies blocking one arm to observe effects on the other remains scarce and represents a critical research gap. A concise summary of these connecting nodes, together with their evidence levels, causal directions, and corresponding research gaps, is provided in **Table 1**.

**Table 1:** Key connecting nodes, evidence levels, and research gaps for the bidirectional interaction.

Connecting node	Representative evidence	Evidence level	Causal direction	Research gap
ROS-NF-κB axis	Hyperglycemia-induced ROS activates NF-κB in gastric mucosa; AG-derived LPS sustains systemic NF-κB Refs (93–98).	Preclinical; observational	DM→AG and AG→DM (bidirectional)	No simultaneous ROS/NF-κB profiling in dual-disease cohorts
PINK1/Parkin mitophagy	High glucose suppresses Parkin via FOXO1; B_12_/iron deficiency impairs mitochondrial function Refs (99–102).	Preclinical	DM→AG and AG→DM (convergent)	No interventional mitophagy studies in comorbid models
Th17/Treg imbalance	STAT3-driven Th17 bias damages both parietal cells and β-cells Refs (90–92).	Preclinical; genetic association	Common soil (shared)	Limited human data on dual-tissue immune profiling
Gastrin-ghrelin axis	Hypergastrinemia activates CCK-BR on β-cells; ghrelin deficiency impairs muscle metabolism Refs (39–48, 64–73).	Preclinical; cross-sectional	DM→AG and AG→DM (bidirectional)	No prospective hormonal intervention trials in AG

**Table 1** summarizes the key molecular nodes connecting the DM→AG and AG→DM directions of the proposed bidirectional cycle. For each node, the table lists representative supporting evidence, the evidence level (preclinical, observational, or genetic association), the inferred causal direction (bidirectional, convergent, or shared common soil), and the principal research gap. Most of the listed evidence is derived from preclinical and observational studies; no node has yet been validated by large-scale prospective interventional trials in comorbid populations, and the proposed causal links therefore require confirmation in future prospective studies.

## 5 Clinical implications and future directions based on mechanism research

In summary, diabetes and atrophic gastritis form a vicious cycle of two-way interaction through multiple mechanisms such as hyperglycemic toxicity, oxidative stress, autophagy dysfunction, microcirculation disorders, and immune disorders. Diabetes damages the gastric mucosal barrier and inhibits cytoprotective mechanisms, thereby accelerating the progression of atrophic gastritis. The imbalance of gastrin-somatostatin, gastrointestinal motility disorder, and intestinal flora disorder caused by atrophic gastritis, in turn, exacerbate abnormal glucose metabolism and insulin resistance (104). A thorough understanding of this interaction provides new targets for clinical intervention, as well as many research gaps and future directions.

### 5.1 Potential therapeutic targets for the interaction mechanism

From the molecular and cellular mechanisms, integrated intervention strategies with dual benefits of “delaying gastric atrophy” and “improving glucose metabolism” can be refined. It should be noted that most of the following targets are currently at preclinical or early translational stages, and their clinical application in diabetic atrophic gastritis requires further validation.

Anti-oxidation and anti-aging strategies. Hyperglycemia-driven oxidative stress and accumulation of AGEs are the core pathological links between them. AGEs not only directly induce apoptosis and autophagic flux disorders in gastric mucosal epithelial cells but also exacerbate chronic inflammation by activating the NF-κB pathway via RAGE. Blocking the AGEs-RAGE axis becomes a key target. Soluble RAGE (sRAGE) acts as a decoy receptor to neutralize circulating AGEs, while AGE cross-linking blockers can reverse the damage caused by established protein cross-linking. Of note, sRAGE and AGE cross-linking blockers remain in preclinical development. The Nrf2 pathway has significant potential to enhance endogenous antioxidant capacity. It not only regulates classical antioxidant enzymes but also helps maintain mitochondrial function and inhibits ferroptosis. Natural products such as curcumin, resveratrol, and astragaloside IV have been shown to alleviate gastric mucosal oxidative damage in a high-glucose environment by activating Nrf2 or directly scavenging free radicals (105); these agents have shown safety in early-phase trials but lack disease-specific efficacy data in diabetic gastropathy. In the future, the strategy of “hormetic effect” with long-term low-dose administration should be explored to activate the adaptive stress response.

Improved mucosal microcirculation and HIF-1α regulation. Diabetic gastric mucosal microangiopathy leads to local ischemia and hypoxia, which are key factors for delayed ulcer healing and atrophy progression. PGE_2_ analogs can directly protect gastric mucosa and improve microcirculation. However, selective COX-2 inhibitors may aggravate gastric ulcers by inhibiting the production of protective prostaglandins, which should be used with caution. HIF-1α is the core transcription factor for cell adaptation to hypoxia. Oxidative stress may impair HIF-1α activity in patients with diabetic gastropathy, leading to down-regulation of VEGF expression and hindering angiogenesis and tissue repair. Prolyl hydroxylase inhibitors, which stabilize HIF-1α under normoxia, have the potential to “mimic hypoxic adaptation” and have been shown to attenuate colitis in animal studies; these agents are approved for anemia but their application in diabetic gastropathy is off-label and requires dedicated clinical trials (106).

Regulation of gastrointestinal hormones and prokinetic interventions. Hypergastrinemia and gastroparesis are common consequences of diabetic autonomic neuropathy. Persistently high gastrin levels stimulate ECL cell proliferation and promote the proliferation and dedifferentiation of gastric mucosal cells via activation of the CCK-2 receptor, thereby accelerating dysplasia. Somatostatin analogs can inhibit gastrin secretion and delay islet cell function decline. In theory, somatostatin analogs can break the chain of “hyperglycemia-gastrin-mucosal remodeling”; however, these agents are available for other indications but have not been systematically evaluated for diabetic atrophic gastritis. Ghrelin agonists improve gastric emptying and increase appetite, but their use requires careful balancing in obese patients with type 2 diabetes. Inhibition of gastric emptying by GLP-1 receptor agonists may exacerbate preexisting gastroparesis and should be selected on an individual basis according to the patient’s phenotype (107).

Adjusting the intestinal microecology. Both of them have an obvious imbalance in intestinal flora. Long-term high blood sugar and acid deficiency promote SIBO, produce large amounts of LPS in the portal vein, induce endotoxin-mediated metabolic disease, intensify systemic inflammation and insulin resistance, and reduce SCFA production by beneficial bacteria, thereby weakening intestinal barrier function. Probiotics, prebiotics, or fecal microbiota transplantation can reshape the microecology, reduce LPS levels, and reduce the load of endogenous AGEs (108). Specific strains targeting the “gut-stomach axis” can efficiently ferment polysaccharides to produce butyrate, which inhibits NF-κB phosphorylation by activating PPARγ, thereby reducing gastric mucosal inflammation, delaying atrophy, and improving glycemic control, making this an integrated intervention with promise.

Targeting autophagy homeostasis and cellular senescence. The autophagic flux is blocked in gastric epithelial cells in a high-glucose/AGEs environment, as evidenced by the accumulation of LC3-II and impaired clearance of p62, leading to the accumulation of damaged mitochondria and misfolded proteins, thereby triggering apoptosis and inflammatory senescence. Rapamycin, as an mTOR inhibitor, can induce autophagy and alleviate diabetes-induced delayed gastric emptying, but long-term use has significant metabolic side effects. Trehalose, as an mTOR-independent autophagy inducer, is safe and has more translational potential, though it remains in early clinical development. Metformin activates autophagy through the AMPK pathway and has a direct protective effect on gastric mucosa; it is the only agent with established clinical safety, but its gastric mucosal protective effect requires prospective confirmation, providing a theoretical basis for the potential “one stone, two birds” effect in diabetic patients with atrophic gastritis (109).

### 5.2 Limitations of current research and future exploration directions

It is important to objectively evaluate the shortcomings of existing research and identify potential breakthroughs at the frontier of the discipline to guide future research directions.

There is a lack of causal evidence from prospective cohorts. Most of the current evidence is predominantly derived from cross-sectional, retrospective, or preclinical studies, which limits the ability to infer definitive causal relationships between diabetes and atrophic gastritis. It is not possible to clarify whether hyperglycemia directly induces gastric mucosal atrophy or whether atrophic gastritis leads to malnutrition and motility disorders that accelerate the progression of diabetes. Furthermore, direct evidence for the closed-loop interaction—such as simultaneous biomarker changes reflecting activation of both pathways in the same patient cohort, or interventional studies showing that blocking one arm ameliorates progression of the other—remains scarce. A large-sample prospective cohort, with dynamic monitoring of glucose metabolism indicators, gastroscopy and assessment of mucosal pathological changes, and collection of serum for biobanking to detect gastrin 17, PGR, anti-parietal cell antibody, AGEs, sRAGE, etc., is urgently needed. Dual-biomarker studies that simultaneously measure gastric atrophy markers and metabolic indicators in the same cohort would be particularly valuable for validating the closed-loop model. Through long-term follow-up, we can establish time-series associations and identify subgroups at high risk of conversion, thereby providing high-level evidence to inform the development of integrated screening guidelines.

Biomarker Development and Validation. At present, there are no specific, noninvasive biomarkers that dynamically reflect the interaction. Serum PGR is susceptible to PPI and H. pylori eradication status. Circulating miRNAs can regulate autophagy-related genes and persist in exosomes, which is expected to serve as a liquid biopsy indicator of autophagic activity in the gastric mucosa (110). Methylation markers change in gastric precancerous lesions. A high-glucose environment can induce the expression of DNA methyltransferases, suggesting that the methylation profile of free DNA in peripheral blood or gastric juice may serve as a warning signal. In the future, case-control studies are needed to verify its diagnostic efficacy.

In-depth exploration of animal models and organoid platforms. Existing animal models have critical limitations that must be acknowledged (1): STZ-induced models primarily simulate type 1 diabetes with acute β-cell destruction, which does not recapitulate the chronic insulin-resistant state of type 2 diabetes that accounts for >90% of clinical cases (2); chemical-induced gastric ulcer models focus on acute injury and repair rather than the chronic progressive atrophy seen in human AG (3); no currently available model simultaneously recapitulates both diabetes and atrophic gastritis with their full clinical comorbidities, making it difficult to study the bidirectional interaction (4); species differences in gastric anatomy (e.g., rodents lack a distinct antrum-body boundary) and H. pylori infection biology limit direct translatability. For future translational validation, we propose explicit steps: first, construct dual-disease models using spontaneous type 2 diabetes (db/db, ZDF rats) combined with chronic H. pylori (SS1 strain) infection or high-fat diet plus low-dose STZ plus high-salt/high-AGE diet; second, validate key mechanistic findings in patient-derived gastric mucosal organoids with fluorescent autophagy reporters (mCherry-GFP-LC3); third, use microfluidic chip platforms for high-throughput screening of candidate drugs such as Nrf2 activators, HIF-1α stabilizers, and mTOR-independent autophagy inducers; fourth, prioritize targets that show efficacy in at least two independent model systems before advancing to clinical trials. At the same time, a patient-derived gastric mucosal organoid was used, and a fluorescent autophagy reporter system (mCherry-GFP-LC3) was introduced to observe the differentiation and autophagic flux of gastric epithelial stem cells in real time. Combined with the microfluidic chip, candidate drugs such as Nrf2 activators, HIF-1α stabilizers, and mTOR-independent autophagy inducers can be screened in high throughput.

From “sugar-stomach dialogue” to precise classification and intervention. Current clinical management separates the two and lacks integration. We propose a protocol-driven screening framework (1): For all patients with type 2 diabetes duration >5 years or HbA1c >7.5%, screen serum pepsinogen ratio (PGR), gastrin-17, anti-parietal cell antibody (APCA), and homocysteine annually (2); Patients with PGR <3 or gastrin-17 > upper limit of normal should be referred for gastroscopy with biopsy (3); For patients with confirmed atrophic gastritis, assess fasting glucose, HbA1c, and islet autoantibodies at diagnosis and every 6 months thereafter. The precise phenotypic classification of “diabetic gastropathy” is proposed, including the atrophy-immune-dominant type (gastric body atrophy, hypergastrinemia, antibody-positive, vitamin B_12_ deficiency), the dynamic-metabolism-dominant type (gastroparesis, postprandial fullness, early satiety, with peripheral neuropathy), and the mixed type. Based on this, a risk model including PGR, gastrin 17, anti-Hp antibody, anti-parietal cell antibody, HbA1c, and disease course was developed, and regular gastroscopy was recommended for high-risk patients. Regarding treatment, the establishment of an “endocrine-digestive combined clinic”, staging classification, intensive glucose control for atrophy type, and supplementation with vitamin B_12_, iron, and calcium, with PPI use to be used with caution. Dynamic type: choose prokinetic drugs that do not affect blood glucose, consider ghrelin agonists, evaluate the side effects of GLP-1 receptor agonists, and switch to SGLT2 inhibitors when necessary. Through integrated strategies to achieve gastrointestinal protection during the whole course of diabetes, block the “sugar-stomach” vicious cycle, reduce the risk of gastric cancer, and improve the quality of life.

## 6 Conclusion

The pathological association between diabetes and atrophic gastritis is not merely a coincidence of comorbidities but may represent a bidirectional vicious cycle driven by the intertwining of multiple molecular events. The graphical illustration of the above mechanisms is shown in **Figures 1** and **2**. From the perspective of diabetes promoting atrophic gastritis, long-term hyperglycemia exerts pathological pressure through three core pathways. High glucose toxicity directly induces local oxidative stress and accumulation of AGEs in the gastric mucosa, activates the NF-κB signaling axis, causes excessive apoptosis of gastric epithelial cells and disordered autophagic flux, and leads to decreased glandular density and mucosal thinning. Diabetic microangiopathy and autonomic nerve dysfunction jointly cause chronic gastric mucosal ischemia, hypoperfusion, and motility disorders. Delayed gastric emptying and bile reflux continue to damage the mucosa. At the same time, the reduction of interstitial cells of Cajal weakens the self-cleaning and repair ability of the stomach. The high-glucose microenvironment enhances the efficiency of H. pylori adhesion and colonization, which, combined with diabetes-related immunodeficiency, leads to persistent infection and amplifies the Correa cascade toward atrophy and metaplasia. These three pathways reinforce one another and constitute the molecular pathology of the gastric mucosa, from inflammation to atrophy.

On the contrary, atrophic gastritis has the opposite effect on glucose metabolism. The progressive loss of parietal cells impairs the absorption of vitamin B_12_ and iron, leading to hyperhomocysteinemia and mitochondrial respiratory chain dysfunction. The former aggravates peripheral insulin resistance, and the latter impairs the GSIS ability of β-cells. Atrophic gastritis also remodels the gastrointestinal hormone network: extreme hypergastrinemia accelerates β-cell dedifferentiation and functional exhaustion through persistent hyperstimulation of CCK-B receptors, while significant ghrelin deficiency releases the fine inhibition of insulin secretion and weakens skeletal muscle metabolic reserve. At the same time, SIBO induced by low gastric acid produces a large amount of LPS, which enters the portal vein blood and causes metabolic endotoxemia, activates systemic low-grade inflammation, impairs the function of GLP-1/GIP axis, and worsens postprandial glucose control. These pathways together constitute a reverse pathological closed loop of “gastric mucosal atrophy - nutrient deprivation, hormone disorder, flora imbalance - aggravation of insulin resistance and β-cell function decline - accelerated progression of diabetes”.

In conclusion, diabetes and atrophic gastritis may form a bidirectional closed loop of “hyperglycemic toxicity - neurovascular injury, H. pylori susceptibility, hormone network imbalance - gastric mucosal atrophy - micronutrient deficiency, flora imbalance, new hormone disorder - deterioration of blood glucose and aggravation of complications.” At present, research has progressed from the description of phenomena to molecular dialogue, but there remains a gap in evidence for clinical translation. Most mechanistic links remain to be verified by prospective clinical studies, and the causal direction and temporal sequence require confirmation in well-designed longitudinal cohorts. Prospective cohorts are lacking to clarify the causal timing; non-invasive, disease-specific biomarkers are lacking to dynamically monitor the strength of interaction; and animal models and organoid platforms need to simulate the complex phenotype of dual diseases. In the future, the concept of “glucose-gastrotube co-administration” should be promoted, and serum pepsinogen ratio, gastrin 17, anti-parietal cell antibody, and homocysteine should be included in the routine assessment of diabetes, and integrated interventions such as anti-oxidation, anti-aging, autophagy regulation, microecology remodeling, and personalized prokinetics should be implemented on the basis of accurate classification. Only by breaking the organ barriers can this vicious cycle of mutual promotion be broken and the risk of gastric precancerous lesions and end-stage glucose-metabolism decompensation be reduced.

## References

[1] Shah SC Piazuelo MB Kuipers EJ Li D . Aga clinical practice update on the diagnosis and management of atrophic gastritis: Expert review. Gastroenterology. (2021) 161:1325–1332.e7. doi: 10.1053/j.gastro.2021.06.078 34454714 PMC8740554

[2] Storman M Czupryniak L . Type 2 diabetes and gastrointestinal cancers: Risk associations and awareness of screening challenges. Cancers (Basel). (2025) 17:2989. doi: 10.3390/cancers17182989 41008833 PMC12468202

[3] Abbasi E Khodadadi I . High-fat diet may increase the risk of insulin resistance by inducing dysbiosis. Metabol Open. (2025) 27:100381. doi: 10.1016/j.metop.2025.100381 40741424 PMC12309925

[4] Dias AS Llesuy S Marroni CA Marroni N . Alterações gastrointestinais no diabetes mellitus: estresse oxidativo e fluxo sangüíneo da artéria mesentérica--estudo experimental [Gastrointestinal alterations in diabetes mellitus: oxidative stress and blood flow in the superior mesenteric artery--experimental study. Arq Gastroenterol. (2004) 41:108–13. doi: 10.1590/s0004-28032004000200008 15543384

[5] Szlachcic A Krzysiek-Maczka G Pajdo R Targosz A Magierowski M Jasnos K . The impact of asymmetric dimethylarginine (ADAMA), the endogenous nitric oxide (NO) synthase inhibitor, to the pathogenesis of gastric mucosal damage. Curr Pharm Des. (2013) 19:90–7. doi: 10.2174/13816128130113 22950506

[6] Tunali S Yanardag R . Effect of vanadyl sulfate on the status of lipid parameters and on stomach and spleen tissues of streptozotocin-induced diabetic rats. Pharmacol Res. (2006) 53:271–7. doi: 10.1016/j.phrs.2005.12.004 16431126

[7] Mahavadi S Sriwai W Manion O Grider JR Murthy KS . Diabetes-induced oxidative stress mediates upregulation of RhoA/Rho kinase pathway and hypercontractility of gastric smooth muscle. PloS One. (2017) 12:e0178574. doi: 10.1371/journal.pone.0178574 28678840 PMC5497948

[8] Magierowski M Jasnos K Brzozowska I Drozdowicz D Sliwowski Z Nawrat E . Melatonina jako czynnik leczniczy wzgledem wrzodów zoładka w warunkach eksperymentalnej cukrzycy [Melatonin as a therapeutic factor in gastric ulcer healing under experimental diabetes. Przegl Lek. (2013) 70:942–6. 24697035

[9] Bosma KJ Andrei SR Katz LS Smith AA Dunn JC Ricciardi VF . Pharmacological blockade of the EP3 prostaglandin E2 receptor in the setting of type 2 diabetes enhances β-cell proliferation and identity and relieves oxidative damage. Mol Metab. (2021) 54:101347. doi: 10.1016/j.molmet.2021.101347 34626853 PMC8529552

[10] Rojas A Lindner C Schneider I González I Morales MA . Contributions of the receptor for advanced glycation end products axis activation in gastric cancer. World J Gastroenterol. (2023) 29:997–1010. doi: 10.3748/wjg.v29.i6.997 36844144 PMC9950863

[11] Wada K Nakashima Y Yamakawa M Hori A Seishima M Tanabashi S . Dietary advanced glycation end products and cancer risk in Japan: From the Takayama study. Cancer Sci. (2022) 113:2839–48. doi: 10.1111/cas.15455 35662347 PMC9357612

[12] Yu W Chen G Yan J Wang X Zhu Y Zhu L . Single-cell sequencing analysis reveals gastric cancer microenvironment cells respond vastly different to oxidative stress. J Transl Med. (2022) 20:250. doi: 10.1186/s12967-022-03411-w 35659682 PMC9164398

[13] Yang PJ Tseng PH Chang K Chao C Wei J Yang W . Reduced gastric mucosal innervation in obesity: A biomarker predicting remission of diabetes after bariatric surgery. Obes (Silver Spring). (2025) 33:2151–9. doi: 10.1002/oby.70012 40898694

[14] Wei C Sun H Liu S Hu J Cao B . A nomogram for predicting survival based on hemoglobin A1c and circulating tumor cells in advanced gastric cancer patients receiving immunotherapy. Int Immunopharmacol. (2024) 142:113239. doi: 10.1016/j.intimp.2024.113239 39306892

[15] Watanabe M Watari I Akakura M Jiratchaya S Ono T . Alteration in gastric ghrelin expression in a mouse model of type 2 diabetes mellitus. Cureus. (2025) 17:e96035. doi: 10.7759/cureus.96035 41195415 PMC12583231

[16] Eskandaros MS . Antrum preservation versus antrum resection in laparoscopic sleeve gastrectomy with effects on gastric emptying, body mass index, and type II diabetes remission in diabetic patients with body mass index 30-40 kg/m2: a randomized controlled study. Obes Surg. (2022) 32:1412–20. doi: 10.1007/s11695-022-05982-5 35304705 PMC8986727

[17] John JN Gorman S Scales D Gorman J . Online misleading information about women's reproductive health: A narrative review. J Gen Intern Med. (2025) 40:1123–31. doi: 10.1007/s11606-024-09118-6 39511120 PMC11968640

[18] Zheng J Gao Y Xie SH Santoni G Lagergren J . Haemoglobin A1c and serum glucose levels and risk of gastric cancer: a systematic review and meta-analysis. Br J Cancer. (2022) 126:1100–7. doi: 10.1038/s41416-021-01693-3 35027671 PMC8979989

[19] Hu A Schmidt MHH Heinig N . Microglia in retinal angiogenesis and diabetic retinopathy. Angiogenesis. (2024) 27:311–31. doi: 10.1007/s10456-024-09911-1 38564108 PMC11303477

[20] Wu X Yang Z Li Z Yang L Wang X Wang C . Increased expression of hypoxia inducible factor-1 alpha and vascular endothelial growth factor is associated with diabetic gastroparesis. BMC Gastroenterol. (2020) 20:216. doi: 10.1186/s12876-020-01368-y 32650726 PMC7350597

[21] Konturek PC Brzozowski T Burnat G Szlachcic A Koziel J Kwiecien S . Gastric ulcer healing and stress-lesion preventive properties of pioglitazone are attenuated in diabetic rats. J Physiol Pharmacol. (2010) 61:429–36. 20814070

[22] Andersson M Ågren A Henriksson P Wallén H Thorell A . Influence of bariatric surgery on endothelial and glycocalyx biomarkers in obesity and type 2 diabetes. J Clin Endocrinol Metab. (2025) 110:e3193–202. doi: 10.1210/clinem/dgaf092 39943818 PMC12448601

[23] Kornum DS Krogh K Keller J Malagelada C Drewes AM Brock C . Diabetic gastroenteropathy: a pan-alimentary complication. Diabetologia. (2025) 68:905–19. doi: 10.1007/s00125-025-06365-y 39934370 PMC12021976

[24] Hosking DJ Moody F Stewart IM Atkinson M . Vagal impairment of gastric secretion in diabetic autonomic neuropathy. Br Med J. (1975) 2:588–90. doi: 10.1136/bmj.2.5971.588 1131624 PMC1673512

[25] Maalouf E Chai X Patraw JA Cipriani G Linden DR Bernard CE . Changes in interstitial cells of Cajal calcium activity in a diabetic mouse model of delayed gastric emptying. J Physiol. (2026) 604(14):5800–16. doi: 10.1113/JP290718 42218355 PMC13370688

[26] Shargo R Poonen-Honig I Thélin C DuCoin CG Mhaskar R Docimo S . Dumping syndrome and bile acid reflux following pyloroplasty and gastric peroral endoscopic myotomy (G-POEM) for refractory gastroparesis: a systematic review. Cureus. (2025) 17:e79056. doi: 10.7759/cureus.79056 40104464 PMC11914853

[27] Grover M Farrugia G Lurken MS Bernard CE Faussone-Pellegrini MS Smyrk TC . Cellular changes in diabetic and idiopathic gastroparesis. Gastroenterology. (2011) 140:1575–1585.e8. doi: 10.1053/j.gastro.2011.01.046 21300066 PMC3081914

[28] Eisenman ST Gibbons SJ Verhulst PJ Cipriani G Saur D Farrugia G . Tumor necrosis factor alpha derived from classically activated "M1" macrophages reduces interstitial cell of Cajal numbers. Neurogastroenterol Motil. (2017) 29:10.1111/nmo.12984. doi: 10.1111/nmo.12984 27781339 PMC5367986

[29] Choi KM Kashyap PC Dutta N Stoltz GJ Ordog T Shea-Donohue T . CD206-positive M2 macrophages that express heme oxygenase-1 protect against diabetic gastroparesis in mice. Gastroenterology. (2010) 138:2399–2409.e1. doi: 10.1053/j.gastro.2010.02.014 20178793 PMC2883675

[30] Zheng H Liu YJ Chen ZC Fan GQ . miR-222 regulates cell growth, apoptosis, and autophagy of interstitial cells of Cajal isolated from slow transit constipation rats by targeting c-kit. Indian J Gastroenterol. (2021) 40:198–208. doi: 10.1007/s12664-020-01143-7 33792838

[31] Zhang YX Zhang YJ Li M Tian JX Tong XL . Common pathophysiological mechanisms and treatment of diabetic gastroparesis. J Neurogastroenterol Motil. (2024) 30:143–55. doi: 10.5056/jnm23100 38576367 PMC10999838

[32] Kløve S Stinson SE Romme FO Butt J Graversen KB Lund MAV . Helicobacter pylori seropositivity associates with hyperglycemia, but not obesity, in Danish children and adolescents. BMC Med. (2024) 22:379. doi: 10.1186/s12916-024-03591-w 39256870 PMC11389555

[33] Salardi S Cacciari E Menegatti M Landi F Mazzanti L Stella FA . Helicobacter pylori and type 1 diabetes mellitus in children. J Pediatr Gastroenterol Nutr. (1999) 28:307–9. doi: 10.1097/00005176-199903000-00017 10067733

[34] Pathirana R Capitani N McCrory C Della Bella C Stuart I Gilmore W . Helicobacter pylori induces the production of interleukin-37 to promote broad immunosuppression and enhance colonization. Gut Microbes. (2026) 18:2618860. doi: 10.1080/19490976.2026.2618860 41689434 PMC12915765

[35] Pacheco A Diedisheim M Goulvestre C Alexandre-Heymann L Mallone R Dubois-Laforgue D . Screening for autoimmune atrophic gastritis by serum gastrin measurement in subjects with type 1 diabetes. Diabetes Metab. (2025) 51:101640. doi: 10.1016/j.diabet.2025.101640 40113012

[36] Wang L Cao ZM Zhang LL Dai XC Liu ZJ Zeng YX . Helicobacter pylori and autoimmune diseases: Involving multiple systems. Front Immunol. (2022) 13:833424. doi: 10.3389/fimmu.2022.833424 35222423 PMC8866759

[37] Marzinotto I Dottori L Baldaro F Dilaghi E Brigatti C Bazzigaluppi E . Intrinsic factor autoantibodies by luminescent immuno-precipitation system in patients with corpus atrophic gastritis. J Transl Autoimmun. (2021) 4:100131. doi: 10.1016/j.jtauto.2021.100131 35005595 PMC8716657

[38] Gonzalez A Latorre G Paredes L Montoya L Maquilon S Shah SC . Mucocutaneous manifestations in autoimmune gastritis: a prospective case-control study. Am J Gastroenterol. (2021) 116:2374–84. doi: 10.14309/ajg.0000000000001501 34665156 PMC8863405

[39] Taylor L McCaddon A Wolffenbuttel BHR . Creating a framework for treating autoimmune gastritis-the case for replacing lost acid. Nutrients. (2024) 16:662. doi: 10.3390/nu16050662 38474790 PMC10934127

[40] Kumar M Chapman A Javed S Alam U Malik RA Azmi S . The investigation and treatment of diabetic gastroparesis. Clin Ther. (2018) 40:850–61. doi: 10.1016/j.clinthera.2018.04.012 29748143

[41] Evans-Molina C Pettway YD Saunders DC Sharp SA Bate TS Sun H . Heterogeneous endocrine cell composition defines human islet functional phenotypes. Nat Commun. (2026) 17:4223. doi: 10.1038/s41467-026-70689-5 42120367 PMC13168233

[42] Tegehall A Korsgren O Ingvast S Gasparyan G Granlund L Lundberg M . Islet delta-cell architecture is remodelled in the human pancreas during type 1 diabetes. Sci Rep. (2025) 15:19776. doi: 10.1038/s41598-025-04471-w 40473678 PMC12141693

[43] Nilsson CI Dumral Ö Sanchez G Xie B Müller A Solimena M . Somatostatin triggers local cAMP and Ca2+signaling in primary cilia to modulate pancreatic β-cell function. EMBO J. (2025) 44:1663–91. doi: 10.1038/s44318-025-00383-7 39939781 PMC11914567

[44] Chinen K Sakata N Yoshimatsu G Nakamura M Kodama S . Therapeutic effects of acylated ghrelin-specific receptor GHS-R1a antagonist in islet transplantation. Sci Rep. (2021) 11:21239. doi: 10.1038/s41598-021-00740-6 34711885 PMC8553779

[45] Ng XW Kong C DiGruccio MR Lee J Piston DW . Role of complexin 2 in the regulation of hormone secretion from the islet of Langerhans. Am J Physiol Endocrinol Metab. (2025) 329:E861–73. doi: 10.1152/ajpendo.00519.2024 41212554 PMC12990977

[46] Hill TG Gao R Benrick A Kothegala L Rorsman N Santos C . Loss of electrical β-cell to δ-cell coupling underlies impaired hypoglycaemia-induced glucagon secretion in type-1 diabetes. Nat Metab. (2024) 6:2070–81. doi: 10.1038/s42255-024-01139-z 39313541 PMC11599053

[47] Mawla AM van der Meulen T Huising MO . Chromatin accessibility differences between alpha, beta, and delta cells identifies common and cell type-specific enhancers. BMC Genomics. (2023) 24:202. doi: 10.1186/s12864-023-09293-6 37069576 PMC10108528

[48] Klein L Ziegler S Gerst F Morgenroth Y Gotkowski K Schöniger E . Explainable AI-based analysis of human pancreas sections identifies traits of type 2 diabetes. Nat Commun. (2026) 17:1558. doi: 10.1038/s41467-026-69295-2 41663377 PMC12894717

[49] Zacharias BS Upendra S Dumbre D . From micro to macro: Understanding the far-reaching consequences of B12 deficiency on women's health. Public Health Nurs. (2025) 42:1115–31. doi: 10.1111/phn.13525 39748484

[50] Al-Samydai A Oraibi A Altamimi S Al-Hussaniy H Zainee H Kareem L . Serum vitamin B12, folic acid, homocysteine, and glycemic control markers in patients with type 2 diabetes by metformin and other oral antidiabetic medications. Georgian Med News. (2025) (362):2–97. 40737654

[51] Maher A Sobczyńska-Malefora A . The relationship between folate, vitamin B12 and gestational diabetes mellitus with proposed mechanisms and foetal implications. J Family Reprod Health. (2021) 15:141–9. doi: 10.18502/jfrh.v15i3.7131 34721605 PMC8536822

[52] Verma A Jaiswal S Gupta S Mittal N Lal M . Association of vitamin B12 deficiency and hyperhomocystinemia with acute coronary syndromes - a case series. Am J Emerg Med. (2025) 94:253.e5–8. doi: 10.1016/j.ajem.2025.04.068 40312197

[53] Qin Y Song Q Jiang X Su Y Chen H Ji X . Correlation between serum vitamin levels and gestational diabetes mellitus. Front Endocrinol (Lausanne). (2025) 16:1569654. doi: 10.3389/fendo.2025.1569654 41127511 PMC12537430

[54] Ramirez-Villalobos MD Monterrubio-Flores E Marquez-Murillo M Alcalde-Rabanal J Shamah-Levy T Perichart-Perera O . Vitamin B12 deficiency, hyperhomocysteinemia, and diabetes as metabolic determinants of cardiovascular risk in Mexican women. Nutrients. (2025) 17:3535. doi: 10.3390/nu17223535 41305587 PMC12655247

[55] Park S Kang S Sol Kim D . Folate and vitamin B-12 deficiencies additively impaire memory function and disturb the gut microbiota in amyloid-β infused rats. Int J Vitam Nutr Res. (2022) 92:169–81. doi: 10.1024/0300-9831/a000624 31841076

[56] Yazidi M Kamoun E Hadj Taieb S Rejeb O Mahjoub S Maryam S . Vitamin B12 deficiency in long-term metformin treated type 2 diabetic patients: Prevalence and risk factors in a Tunisian population. Chronic Illn. (2024) 20:631–9. doi: 10.1177/17423953231184220 37337655

[57] Sakouhi M Matmour D Belakhdar K Kraroubi A . Étude transversale sur les effets indésirables de la chlorhydrate de métformine chez 130 patients diabétiques type 2 admis au centre médical et à la maison du diabète de Sidi Bel-Abbès [cross-sectional study on adverse effects of metformin hydrochloride on 130 patients type 2 diabetic admitted to medical center and diabetes home of Sidi Bel-Abbès. Ann Pharm Fr. (2023) 81:684–95. doi: 10.1016/j.pharma.2022.11.013 36464072

[58] Cooksey RC Jones D Gabrielsen S Huang J Simcox JA Luo B . Dietary iron restriction or iron chelation protects from diabetes and loss of beta-cell function in the obese (ob/ob lep-/-) mouse. Am J Physiol Endocrinol Metab. (2010) 298:E1236–43. doi: 10.1152/ajpendo.00022.2010 20354157 PMC2886527

[59] Inoue Y Nakanishi K Hiraga T Okubo M Murase T Kosaka K . Recovery of pancreatic beta-cell function in hemochromatosis: combined treatment with recombinant human erythropoietin and phlebotomy. Am J Med Sci. (1997) 314:401–2. doi: 10.1097/00000441-199712000-00008 9413346

[60] Fung EB Ahmad T Killilea DW Hussain R Lal A . Zinc supplementation improves markers of glucose homeostasis in thalassaemia. Br J Haematol. (2020) 190:e162–6. doi: 10.1111/bjh.16771 32488893

[61] Bravo L Martínez-López S Sierra-Cinos JL Mateos R Sarriá B . Yerba mate (Ilex paraguariensis St. Hill.) tea may have cardiometabolic beneficial effects in healthy and at-risk subjects: a randomized, controlled, blind, crossover trial in nonhabitual consumers. Mol Nutr Food Res. (2025) 69:e70065. doi: 10.1002/mnfr.70065 40263915 PMC12319497

[62] Grong E Nord C Arbo IB Eriksson M Kulseng BE Ahlgren U . The effect of hypergastrinemia following sleeve gastrectomy and pantoprazole on type 2 diabetes mellitus and beta-cell mass in Goto-Kakizaki rats. J Endocrinol Invest. (2018) 41:691–701. doi: 10.1007/s40618-017-0793-9 29168078

[63] Khan MT Zohair M Khan A Kashif A Mumtaz S Muskan F . From gut to brain: The roles of intestinal microbiota, immune system, and hormones in intestinal physiology and gut-brain-axis. Mol Cell Endocrinol. (2025) 607:112599. doi: 10.1016/j.mce.2025.112599 40482955

[64] Vu JP Wang HS Germano PM Pisegna JR . Ghrelin in neuroendocrine tumors. Peptides. (2011) 32:2340–7. doi: 10.1016/j.peptides.2011.10.006 22041110 PMC6707517

[65] Khan D Vasu S Moffett RC Irwin N Flatt PR . Expression of gastrin family peptides in pancreatic islets and their role in β-cell function and survival. Pancreas. (2018) 47:190–9. doi: 10.1097/MPA.0000000000000983 29329158

[66] Skarbaliene J Rigbolt KT Fosgerau K Billestrup N . In-vitro and in-vivo studies supporting the therapeutic potential of ZP3022 in diabetes. Eur J Pharmacol. (2017) 815:181–9. doi: 10.1016/j.ejphar.2017.09.026 28928089

[67] Hasib A Ng MT Khan D Gault VA Flatt PR Irwin N . Characterisation and antidiabetic utility of a novel hybrid peptide, exendin-4/gastrin/xenin-8-Gln. Eur J Pharmacol. (2018) 834:126–35. doi: 10.1016/j.ejphar.2018.07.027 30025814

[68] Toren E Liu Y Bethea M Wade A Hunter CS . The Ldb1 transcriptional co-regulator is required for establishment and maintenance of the pancreatic endocrine lineage. FASEB J. (2022) 36:e22460. doi: 10.1096/fj.202200410R 35881062 PMC9397370

[69] Hasib A Khan D Craig SL Gault VA Flatt PR Irwin N . Antidiabetic effects and sustained metabolic benefits of sub-chronic co-administration of exendin-4/gastrin and xenin-8-Gln in high fat fed mice. Eur J Pharmacol. (2019) 865:172733. doi: 10.1016/j.ejphar.2019.172733 31614140

[70] Feng T Yin W Picoli CC Liu L Le PT Liu Z . Intact mTOR signaling in gastric X/A-like cells is required for bone homeostasis. Front Endocrinol (Lausanne). (2026) 17:1763507. doi: 10.3389/fendo.2026.1763507 42039138 PMC13106587

[71] Suzuki H Masaoka T Hosoda H Nomura S Ohara T Kangawa K . Plasma ghrelin concentration correlates with the levels of serum pepsinogen I and pepsinogen I/II ratio--a possible novel and non-invasive marker for gastric atrophy. Hepatogastroenterology. (2004) 51:1249–54. 15362725

[72] Tuero C Rodríguez A Ramírez B Catalán V Cienfuegos JA Gómez-Ambrosi J . One anastomosis mini-gastric bypass decreases desacyl ghrelin and improves metabolic profile in male rats with diet-induced obesity. J Nutr Biochem. (2026) 147:110124. doi: 10.1016/j.jnutbio.2025.110124 41005575

[73] Polishchuk H Guzik K Kantyka T . Beyond hunger: The structure, signaling, and systemic roles of ghrelin. Int J Mol Sci. (2025) 26:10996. doi: 10.3390/ijms262210996 41303478 PMC12651995

[74] Corleto VD Festa S Di Giulio E Annibale B . Proton pump inhibitor therapy and potential long-term harm. Curr Opin Endocrinol Diabetes Obes. (2014) 21:3–8. doi: 10.1097/MED.0000000000000031 24310148

[75] Bures J Cyrany J Kohoutova D Förstl M Rejchrt S Kvetina J . Small intestinal bacterial overgrowth syndrome. World J Gastroenterol. (2010) 16:2978–90. doi: 10.3748/wjg.v16.i24.2978 20572300 PMC2890937

[76] Crasan IM Tanase M Delia CE Gradisteanu-Pircalabioru G Cimpean A Ionica E . Metaflammation's role in systemic dysfunction in obesity: A comprehensive review. Int J Mol Sci. (2025) 26:10445. doi: 10.3390/ijms262110445 41226484 PMC12607572

[77] Dukowicz AC Lacy BE Levine GM . Small intestinal bacterial overgrowth: a comprehensive review. Gastroenterol Hepatol (N Y). (2007) 3:112–22. PMC3099351 21960820

[78] Long J Zhao J Gu X Huang C . Post-transplant diabetes mellitus after kidney transplantation: pathogenesis, risk factors, and management strategies. Front Endocrinol (Lausanne). (2026) 17:1829579. doi: 10.3389/fendo.2026.1829579 42130741 PMC13167200

[79] Liu Y Wang Y Teng H Nie W Qiao X Shen Y . From gut microbiota metabolism to microvascular injury: Exploring the role and mechanisms of gut microbiota in obesity-induced coronary microcirculation dysfunction. Virulence. (2026) 17:2670787. doi: 10.1080/21505594.2026.2670787 42228778 PMC13232876

[80] Mehboodi M Namdari MP Abdollahi Z Mobarezi Z Kiani M Chamani F . The relationship between increased levels of microbiota-derived lipopolysaccharide in obesity and the pathophysiology of neurodegenerative diseases. Microb Pathog. (2025) 207:107905. doi: 10.1016/j.micpath.2025.107905 40683546

[81] Shahamati D Akhavan NS Rosenkranz SK . Postprandial inflammation in obesity: Dietary determinants, adipose tissue dysfunction and the gut microbiome. Biomolecules. (2025) 15:1516. doi: 10.3390/biom15111516 41301434 PMC12650295

[82] Crnčević N Hukić M Deumić S Selimagić A Dozić A Gavrankapetanović I . Gastrointestinal tract microbiome effect and role in disease development. Diseases. (2022) 10:45. doi: 10.3390/diseases10030045 35892739 PMC9326677

[83] Phuah P Norton M Cheng S Roberts AG Pirri D Meyer L . The microbial tryptophan metabolite indole acts on the gastrointestinal tract to improve glucose homeostasis in a mouse model of diabetes by enhancing GLP-1 secretion and L cell differentiation. Diabetologia. (2026) 69:1659–74. doi: 10.1007/s00125-026-06688-4 41776124 PMC13109135

[84] Jing M Jiang Y . Microbiome-mediated crosstalk between T2DM and MASLD: a translational review focused on function. Front Endocrinol (Lausanne). (2025) 16:1677175. doi: 10.3389/fendo.2025.1677175 41334445 PMC12665569

[85] Liu Y Wang Y Teng H Nie W Qiao X Shen Y . From gut microbiota metabolism to microvascular injury: Exploring the role and mechanisms of gut microbiota in obesity-induced coronary microcirculation dysfunction. Virulence. (2026) 17:2670787. doi: 10.1080/21505594.2026.2670787 PMC13232876 42228778

[86] Monsalve FA Fernández-Tapia B Arriagada OC González DR Delgado-López F . Obesity and depression: A pathophysiotoxic relationship. Int J Mol Sci. (2025) 26:11590. doi: 10.3390/ijms262311590 41373743 PMC12692040

[87] Laisk T Lepamets M Koel M Abner E Estonian Biobank Research Team . Genome-wide association study identifies five risk loci for pernicious anemia. Nat Commun. (2021) 12:3761. doi: 10.1038/s41467-021-24051-6 34145262 PMC8213695

[88] Kozhakhmetova A Wyatt RC Caygill C Williams C Long AE Chandler K . A quarter of patients with type 1 diabetes have co-existing non-islet autoimmunity: the findings of a UK population-based family study. Clin Exp Immunol. (2018) 192:251–8. doi: 10.1111/cei.13115 29431870 PMC5980499

[89] Zhang A Niu L Ni Y Liu W Gao X Chang L . STAT3 inhibition mitigates experimental autoimmune gastritis by restoring Th17/Treg immune balance. Immunol Res. (2025) 73:90. doi: 10.1007/s12026-025-09643-4 40471463

[90] Lenti MV Miceli E Lahner E Natalello G Massironi S Schiepatti A . Distinguishing features of autoimmune gastritis depending on previous Helicobacter pylori infection or positivity to anti-parietal cell antibodies: Results from the Autoimmune gastRitis Italian netwOrk Study grOup (ARIOSO). Am J Gastroenterol. (2024) 119:2408–17. doi: 10.14309/ajg.0000000000002948 38976374

[91] Pilli T Dalmazio G Porcelli B Cantara S Tabucchi A Pini A . Screening of organ-specific autoantibodies in a large cohort of patients with autoimmune thyroid diseases. Thyroid. (2021) 31:1416–23. doi: 10.1089/thy.2021.0170 34281356

[92] Chiaro A Parrinello F Marazzi C Zucconi A Bassi M Calevo MG . Autoimmune gastritis in children: A prospective cohort with serologic screening in at-risk patients. J Pediatr Gastroenterol Nutr. (2026) 82:1408–15. doi: 10.1002/jpn3.70408 41867096

[93] Ock CY Hong KS Choi KS Chung MH Kim YS Kim JH . A novel approach for stress-induced gastritis based on paradoxical anti-oxidative and anti-inflammatory action of exogenous 8-hydroxydeoxyguanosine. Biochem Pharmacol. (2011) 81:111–22. doi: 10.1016/j.bcp.2010.08.023 20816670

[94] Sampath C Raju AV Freeman ML Srinivasan S Gangula PR . Nrf2 attenuates hyperglycemia-induced nNOS impairment in adult mouse primary enteric neuronal crest cells and normalizes stomach function. Am J Physiol Gastrointest Liver Physiol. (2022) 322:G368–82. doi: 10.1152/ajpgi.00323.2021 35084215 PMC8897013

[95] Li Q Dong Z Lian W Cui J Wang J Shen H . Ochratoxin A causes mitochondrial dysfunction, apoptotic and autophagic cell death and also induces mitochondrial biogenesis in human gastric epithelium cells. Arch Toxicol. (2019) 93:1141–55. doi: 10.1007/s00204-019-02433-6 30903243

[96] Meng S Duan J Zhao J Zhou Z Sun B Xu Y . Impairment of mitochondrial quality control exacerbates diabetes-related atrial fibrillation by cGAS-STING signaling pathway and cardiomyocyte-macrophage crosstalk. Theranostics. (2026) 16:1701–19. doi: 10.7150/thno.124140 41356195 PMC12680528

[97] Liu X Ma Y Wang X Zhang X Shen C Su Y . Cross-regulatory mechanisms linking ferroptosis, epigenetics, and circadian rhythm to mitochondrial quality control in diabetic cardiomyopathy. J Adv Res. (2026) 84:937–63. doi: 10.1016/j.jare.2025.09.046 41038509 PMC13227240

[98] De R Mazumder S Sarkar S Debsharma S Siddiqui AA Saha SJ . Acute mental stress induces mitochondrial bioenergetic crisis and hyper-fission along with aberrant mitophagy in the gut mucosa in rodent model of stress-related mucosal disease. Free Radic Biol Med. (2017) 113:424–38. doi: 10.1016/j.freeradbiomed.2017.10.009 28993273

[99] Wang YH Chang DY Zhao YY Tang SC Zhao MH Chen M . Mitochondrial protein TOMM7 alleviates diabetic kidney disease by regulating mitophagy via intracellular redistribution of phospholipase PLA2G6. Kidney Int. (2026) 109:337–53. doi: 10.1016/j.kint.2025.10.009 41276015

[100] Lee KM Kim J Jung HL Kim YY Lee J Lee YJ . Exploring mitophagy levels in Drosophila Malpighian tubules unveils the pivotal role of mitophagy in kidney function and diabetic kidney disease. Exp Mol Med. (2025) 57:2364–75. doi: 10.1038/s12276-025-01558-2 41131336 PMC12586521

[101] Hu H Ji R Hao Y Liu Z Yang J Cao Y . XIAP-ULK1-mediated mitophagy modulates carnitine metabolism to mitigate diabetic kidney disease. Autophagy. (2026) 22:207–28. doi: 10.1080/15548627.2025.2581214 41139215 PMC12758225

[102] Yang H Li Y Hu B . Potential role of mitochondria in gastric cancer detection: Fission and glycolysis. Oncol Lett. (2021) 21:439. doi: 10.3892/ol.2021.12700 33868477 PMC8045152

[103] Luo L Que M Zeng L Wang X Sun T Zhou Z . Selective autophagy in type 2 diabetes-associated cognitive dysfunction: Insightful mechanisms and therapies. Pharmacol Res. (2025) 220:107947. doi: 10.1016/j.phrs.2025.107947 40940004

[104] Harsch IA Brzozowski T Bazela K Konturek SJ Kukharsky V Pawlik T . Impaired gastric ulcer healing in diabetic rats: Role of heat shock protein, growth factors, prostaglandins and proinflammatory cytokines. Eur J Pharmacol. (2003) 481:249–60. doi: 10.1016/j.ejphar.2003.09.019 14642793

[105] Ravella K Yang H Gangula PR . Impairment of gastric nitrergic and NRF2 system in apolipoprotein E knockout mice. Dig Dis Sci. (2012) 57:1504–9. doi: 10.1007/s10620-012-2070-2 22302246 PMC3677538

[106] Hari S Burns GL Hoedt EC Keely S Talley NJ . Eosinophils, hypoxia-inducible factors, and barrier dysfunction in functional dyspepsia. Front Allergy. (2022) 3:851482. doi: 10.3389/falgy.2022.851482 35769556 PMC9234913

[107] Fowler S Hoedt EC Talley NJ Keely S Burns GL . Circadian rhythms and melatonin metabolism in patients with disorders of gut-brain interactions. Front Neurosci. (2022) 16:825246. doi: 10.3389/fnins.2022.825246 35356051 PMC8959415

[108] Burns GL Talley NJ Keely S . Immune responses in the irritable bowel syndromes: Time to consider the small intestine. BMC Med. (2022) 20:115. doi: 10.1186/s12916-022-02301-8 35354471 PMC8969236

[109] Zha T Ding Y Xu X Zhang Y Guo J Ge H . The oral-gut axis in chronic atrophic gastritis: Current perspectives and integrated strategies. Front Immunol. (2026) 16:1699501. doi: 10.3389/fimmu.2025.1699501 41583463 PMC12823898

[110] Elshazly SM Abd El Motteleb DM Ibrahim IAAE . Hesperidin protects against stress induced gastric ulcer through regulation of peroxisome proliferator activator receptor gamma in diabetic rats. Chem Biol Interact. (2018) 291:153–61. doi: 10.1016/j.cbi.2018.06.027 29944876

